# In Situ Ligation of High‐ and Low‐Affinity Ligands to Cell Surface Receptors Enables Highly Selective Recognition

**DOI:** 10.1002/advs.201700147

**Published:** 2017-07-28

**Authors:** Misako Taichi, Shogo Nomura, Ikuhiko Nakase, Rie Imamaki, Yasuhiko Kizuka, Fumi Ota, Naoshi Dohmae, Shinobu Kitazume, Naoyuki Taniguchi, Katsunori Tanaka

**Affiliations:** ^1^ Biofunctional Synthetic Chemistry Laboratory RIKEN Hirosawa Wako‐shi Saitama 351‐0198 Japan; ^2^ Nanoscience and Nanotechnology Research Center Research Organization of the 21st Century Osaka Prefecture University 1‐2 Gakuen‐cho, Naka Sakai Osaka 599‐8570 Japan; ^3^ Disease Glycomics Team Global Research Center RIKEN‐Max Planck Joint Research Center for System Chemical Biology RIKEN, 2‐1 Hirosawa Wako‐shi Saitama 351‐0198 Japan; ^4^ Biomolecular Characterization Unit RIKEN Center for Sustainable Resource Science 2‐1 Hirosawa Wako‐shi Saitama 351‐0198 Japan; ^5^ Biofunctional Chemistry Laboratory A. Butlerov Institute of Chemistry Kazan Federal University 18 Kremlyovskaya street Kazan 420008 Russia; ^6^ JST‐PRESTO 2‐1 Hirosawa Wako‐shi Saitama 351‐0198 Japan

**Keywords:** cell imaging, cell surfaces, glycan, in situ ligation

## Abstract

This paper reports an entirely unexplored concept of simultaneously recognizing two receptors using high‐ and low‐affinity ligands through ligating them in situ on the target cell surface. This de novo approach is inspired by the pretargeting strategy frequently applied in molecular imaging, and has now evolved as the basis of a new paradigm for visualizing target cells with a high imaging contrast. A distinct advantage of using a labeled low‐affinity ligand such as glycan is that the excess labeled ligand can be washed away from the cells, whereas the ligand bound to the cell, even at the milli molar affinity level, can be anchored by a bioorthogonal reaction with a pretargeted high‐affinity ligand on the surface. Consequently, nonspecific background is minimized, leading to improved imaging contrast. Importantly, despite previously unexplored for molecular imaging, a notoriously weak glycan/lectin interaction can now be utilized as a highly selective ligand to the targets.

Molecular imaging research has focused on noninvasively analyzing the molecular kinetics in small animals for use in diagnostic applications.^1^, ^2^, ^3^, ^4^, ^5^, ^6^, ^7^ In vivo information about biologically active small molecules and biomolecules, such as the localization or expression levels of target receptors, may be readily imaged using fluorescence or radionuclide‐based detection. Although many promising tracers can target specific organs or tumors,^8^, ^9^, ^10^, ^11^, ^12^, ^13^ the selectivity and specificity of these tracers toward target cells must be improved. For example, RGD peptides, high‐affinity ligands of α_V_β_3_ integrins,^9^, ^14^ which are cell adhesion molecules that are highly expressed in tumor vasculature^11^ are widely used to image breast,^15^ brain,^16^ and lung^17^ cancers in small animal models; however, RGD peptides often provide poor imaging contrast because the integrins are also expressed on other endothelial cells. The RGD peptides act as “strongly” interacting integrin ligands that nonselectively bind to the various integrins and are rapidly captured by the cells via receptor‐mediated endocytosis, which decreases the tumor/background signal ratio.

While most studies have focused on optimizing ligands with a high affinity and selectivity to the cell surface targets, we envisioned a new imaging approach that utilized one high‐ and one low‐affinity ligand targeted to independent receptors on a target cell surface. This approach was inspired by the pretargeted method used frequently in the molecular imaging field.^18^, ^19^, ^20^, ^21^, ^22^, ^23^ The concept is schematically presented in **Figure** **1**.

**Figure 1:**
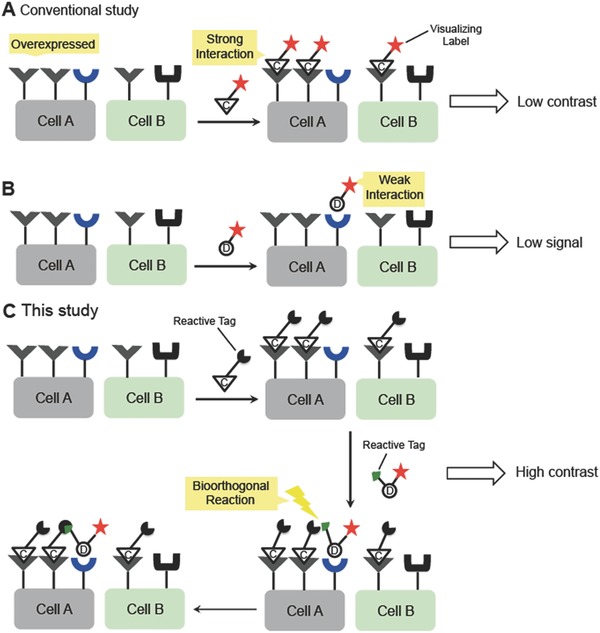
Schematic diagram of cell imaging methods involving high‐ and low‐affinity ligands to cell surface receptors. A) Conventional labeling method using high‐affinity ligand C. B) Labeling method using a low‐affinity ligand D. C) Labeling method using both high‐ and low‐affinity ligands and a bioorthogonal reaction on the cell surface, as reported in this study.

A simplified model was proposed in which various surface receptors are expressed on cells A and B. Cell A may be selectively visualized by applying the fluorescently labeled probe C, which shows a high affinity toward a receptor overexpressed on the surface of A, i.e., at *K*_D_ of nano molar level (Figure 1A). The same receptor could also be expressed more or less on the other cell B, as RGD/integrin interactions were observed on both the tumor cells and the common endothelial cells. Although the quantities of the probe C attached to these cells differed and provided an imaging contrast readout, both A and B could simultaneously be visualized, which decreased the imaging contrast. This is a common issue in the application of ligands that strongly interact with a target cell. Alternatively, probe D may be used for imaging, as it interacts specifically with the target cell A; however, the probe D‐receptor binding is very weak, i.e., at *K*_D_ of milli molar level, and the probe does not bind sufficiently to enable cell A visualization (Figure 1B).

We proposed here to use a combination of the strong and the weak ligand/receptor interactions in a pretargeted fashion (Figure 1C). A high‐affinity probe C prepared without an imaging label but instead with a reactive tag was initially pretargeted to the cells A and B. Subsequently, a low‐affinity probe D prepared with both an imaging label and with bioorthogonal functional groups that can react with the pretargeted tag were applied to the cells. The low‐affinity probe D bound to the receptor very weakly and was washed away immediately from the cell surface; however, binding of probe D to the pretargeted cell selectively anchored the probe to the cell through the bioorthogonal reaction, which was facilitated by the proximal effects between the two surface receptors. The orchestration of strong and weak ligand/receptor interactions and the in situ click conjugation on the cell surface, thus, achieved a high selectivity and imaging contrast between cells A and B. The binding affinity of the labeled probe D should not be high, because if the ligand D bound strongly to the receptor, it could also bind to other cells expressing this receptor or even bind nonspecifically, thereby reducing the imaging selectivity. Such effects are typical of high‐affinity ligands, as discussed in Figure 1A (also see Figure S17, Supporting Information). The use of the weak interaction to the cell surface receptor is therefore a key point to the success of the approach.

The strong–weak ligand synergetic imaging approach was applied using the peptide and glycan ligands, which are representative high‐ and low‐affinity ligands to cell surface receptors (probes C and D in Figure 1C, respectively). It should be noted here that cell labeling using prereacted peptide–glycan conjugates never produced significant imaging contrast because the interactions between the prelinked molecules and the cell surface were plagued with the problems associated with strong peptide/receptor interactions. The weak interactions derived from the glycan/lectin were negligible. Most previous studies of peptide‐ or antibody‐conjugates that utilized the two interactions failed owing to this drawback. Therefore, the in situ ligation concept described in Figure 1C is very important for exploiting the advantages of both the strong and the weak interactions to realize clear target cell imaging contrast (vide infra).

Here, we report a new method for visualizing target cells with a high imaging contrast based on a combination of high‐ and low‐affinity ligands to cell surface receptors. The efficient contrast agents were readily prepared directly on the target cell through an efficient bioorthogonal reaction facilitated by two proximate surface receptors. This study proved that the weak glycan/lectin interactions, not previously explored in molecular imaging research, efficiently provided highly selective cell recognition under appropriate conditions.

The imaging method was demonstrated using a model system in which human umbilical vein endothelial cells (HUVECs) were selectively imaged. Two characteristic receptors, the α_V_β_3_ integrins and the platelet endothelial cell adhesion molecule (PECAM), are expressed in HUVECs (**Figure** **2**). It is reported that the α_V_β_3_ integrins are associated with PECAM on the same endothelial cell surface in a *cis* manner.^24^, ^25^ We previously developed a variety of dendrimer‐type *N*‐glycoclusters with enhanced affinity toward lectins mediated by multivalency effects, and we successfully demonstrated that the PECAM is a sialic acid binding lectin^25^ that exhibits a high affinity toward the α(2,6)‐disialo‐*N*‐glycan (see the structure shown in Figure 2). As expected the single glycan/lectin interaction affinity was low, and this molecule acted as a low‐affinity ligand to PECAM. The cyclic RGDyK peptide was used as the high‐affinity ligand to the α_V_β_3_ integrins. The strain‐promoted azide‐alkyne cycloaddition (SPAAC) reaction was used as the in situ chemoselective and bioorthogonal reaction between the two ligands, which was activated upon binding to the surface receptors.^26^, ^27^ In this work, dibenzocyclooctyne (DIBO), developed by Boons and co‐workers,^28^ was used as the strained acetylene because this acetylene displays a relatively high reactivity and is readily prepared from a simple starting material. The azide function was introduced onto the cyclic RGDyK peptide (Figure 2, **1a–1d**) through different lengths of the short ethylene glycol linkers to investigate the distance‐dependency of the SPAAC reaction, which dictated the distance between the ligand/receptor complexes that were tolerated in the context of the labeling reaction. DIBO and a fluorescent label, tetramethylrhodamine (TAMRA), were attached to α(2,6)‐disialo‐*N*‐glycan through an appropriate linker (**2a**). To confirm the sialic acid‐dependent interaction with PECAM, the asialoglycan derivative (**2b**) was prepared as a control. The syntheses of these functionalized high‐ and low‐affinity ligands are provided in the Supporting Information.

**Figure 2:**
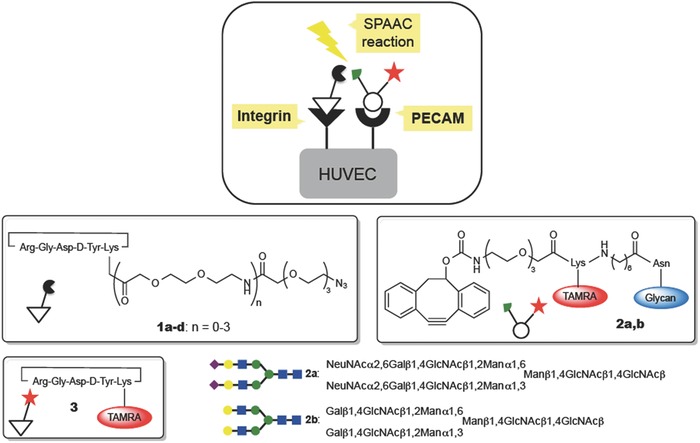
Structures of the functionalized peptide and *N*‐glycan ligands and the symbols used herein.

With the functionalized RGD peptide and *N*‐glycan ligands in hand, we demonstrated the simultaneous targeting of two cell surface receptors by directly linking their high‐ and low‐affinity ligands in situ. This process enabled the selective recognition of HUVECs (**Figure** **3**). The cell incubation conditions were explored to optimize the pretargeting and SPAAC processes. Optimization ensured that the azide group on the pretargeted cyclic RGDyK displayed a sufficiently high clickable reactivity on the cell surface prior to integrin‐mediated endocytosis. Under the optimized conditions, the HUVECs were initially treated with the cyclic RGDyK peptides **1a–1d** (50 µm) for 15 min at room temperature. We have tried to perform the pretargeting with **1a** at 4 °C to inhibit the endocytosis, but affinity of **1a** to integrin could also be significantly reduced, hence the pretargeting conditions described here were found optimal. In order to estimate the amounts of the available azide function on the cell surface prior to the reaction with the second glycan probe **2a**, TAMRA‐labeled cyclic RGDyK peptide 3 (see the structure in Figure 2 and Figure S18, Supporting Information) was treated with the HUVECs under the identical conditions. Cell surface fluorescence, which was colocalized with anti‐PECAM antibody, a representative cell surface marker, was evaluated (Figure S19, Supporting Information); 57% of cell surface TAMRA‐fluorescence was detected out of all fluorescence on a whole cell, which represents the available azide‐tagged **1a** on the cell surface.

**Figure 3:**
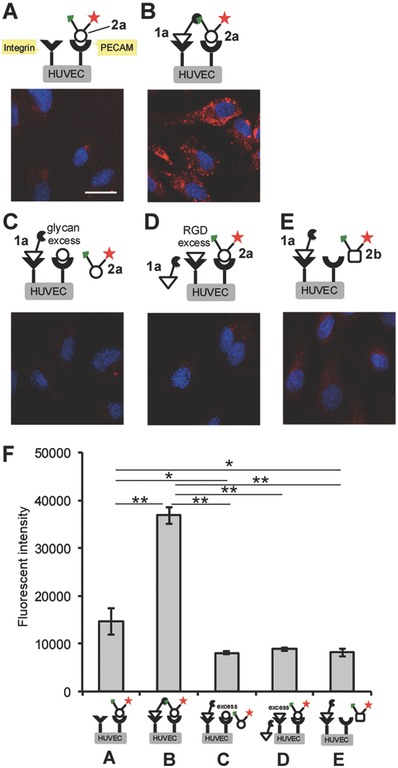
Imaging of HUVECs using both high‐ and low‐affinity ligands and the strain‐promoted azide‐alkyne cycloaddition (SPAAC) reaction. HUVECs were labeled using the following ligand combinations: A) the glycan ligand **2a** alone (red); B) the RGDyK peptide **1a** followed by **2a**; C) the RGDyK peptide **1a** followed by **2a** in the presence of an excess amount of disialo‐*N*‐glycan; D) **1a** in the presence of an excess amount of the RGDyK peptide followed by **2a**; and E) **1a** followed by the asialoglycan ligand **2b** (red). After treatment with the ligands, the cells were fixed and stained with DAPI (blue). The scale bar indicates 20 µm. F) Comparison of the fluorescent intensities measured in (A)–(E). Data are presented as the means ± S.E. [*n* = 10 (10 000 cells × 10), one way ANOVA post hoc Tukey–Kramer's test, *p < 0.01, **p < 0.05].

After washing the cells to remove excess amounts of the cyclic RGDyK peptides, the pretargeted cells were further incubated with the sialoglycan ligand **2a** (50 µm) for 30 min at 4 °C. The cells were then fixed with 4% paraformaldehyde, and the nuclei were stained with 4′,6‐diamidino‐2‐phenylindole (DAPI) prior to confocal microscopy analysis. As a control experiment, the sialoglycan ligand **2a** was incubated without pretargeting the cells using the cyclic RGDyK ligands **1a–1d** under identical conditions.

The cells treated with either the sialoglycan ligand **2a** alone (Figure 3A) and the cells pretargeted with the cyclic RGDyK peptides **1a** (Figure 3B) were compared. The cells pretargeted with **1a** were found to exhibit a threefold higher fluorescence intensity (Figure 3F, also see the schematic comparison of two labeling methods in Figure 1B,C). The direct microscopic imaging of the live cells without fixing the cells, after the treatment with RGDyK peptide **1a** and then sialoglycan **2a**, gave the comparable results with those performed with fixation (Figure S20, Supporting Information). Hence, the fixing procedure does not affect the outcome and the method could be applicable to live cell imaging. Similar results were also obtained by fluorescence activated cell sorting (FACS) analysis (Figure S21, Supporting Information).

The cell surface SPAAC reaction was confirmed by analyzing the clicked products, which were successfully eluted from the cell surface by treatment with KCl‐HCl buffer. Liquid chromatography‐mass spectrometry/mass spectrometry (LC‐MS/MS) profiles of the cell surface products were consistent with those performed with authentic sample (Figures S12–S16, Supporting Information). The yield of the SPAAC reaction on the cell was calculated to be 47% based on the amount of pretargeted RGDyK ligand bound to the cells, and more specifically, 80% based on the RGDyK ligand available on surface (see above discussion for colocalization with PECAM, Figures S18 and S19, Supporting Information). Time dependent fluorescence increase on the whole cells, after treating the pretargeted cells with glycan probe **2a**, provided the kinetics of the cell surface SPAAC reaction (Figure S22, Supporting Information).

It should be noted that the SPAAC reaction conducted in a flask under conditions similar to those applied to the cell‐based experiments in Figure 3B, i.e., 50 µm **1a** and **2a** in phosphate buffered saline (PBS) at 4 °C for 30 min, only yielded trace amounts of the clicked product (Figure S23, Supporting Information). Therefore, the SPAAC reaction was accelerated on the cell surface, i.e., upon ligand binding to the receptors.

Among the cyclic RGDyK peptides **1a–1d**, the peptide that contained the shortest ethylene glycol linker was the most effective for visualizing cells using red fluorescence (Figure S24, Supporting Information). The linker length in **1a** may optimize the distance between two ligands during SPAAC reaction linkage upon binding to the surface receptors.

In order to inhibit the endocytosis of the pretargeted **1a** during the SPAAC processes, we also performed the click reaction after treating the cells with **1a** and then fixing the cells by paraformaldehyde (Figure S25, Supporting Information). A higher amount of pretargeted azide function on RGDyK **1a** could react with the acetylene in glycan **2a**, and the fluorescence contrast was notably improved in comparison with this performed in Figure 3A,B.

The importance of both the strong and the weak ligand/receptor interactions for visualizing the HUVECs was tested by conducting ligand saturation experiments. The cells were treated with excess amounts of the unfunctionalized cyclic RGDyK or the sialoglycan ligands prior to performing the pretargeting procedure; the fluorescence intensities were found to decrease dramatically by 80% and 75%, respectively (Figure 3C,D,F, see also FACS analysis in Figure S21, Supporting Information). Use of the asialoglycan ligand **2b**, which is not a ligand to PECAM, in the SPAAC reaction on the pretargeted cell resulted in a similar decrease of 78% in the fluorescence intensities (Figure 3E,F). These results clearly support the concept, as illustrated in Figure 1C, that each of the ligand/receptor interactions contributed to the SPAAC reaction on the cell surface, thereby enabling the sensitive recognition of the HUVECs that simultaneously expressed the two target receptors on the cell surface.

The selective visualization of the target cells was demonstrated in **Figure** **4**. We initially prepared artificial HUVECs, in which the PECAM expression levels were attenuated by ≈50% through siRNA transfection experiments (see the details in Figure S26, Supporting Information). Not surprisingly, the control HUVEC and PECAM‐knockdown cells did not provide good imaging contrast upon treatment with the TAMRA‐labeled RGDyK 3 (Figure 4A). Despite a decrease in the total fluorescence intensity, our pretargeted method selectively imaged the control HUVECs by implementing a fivefold difference between the fluorescence intensity of the HUVECs over the PECAM‐knockdown cells (Figure 4B).

**Figure 4:**
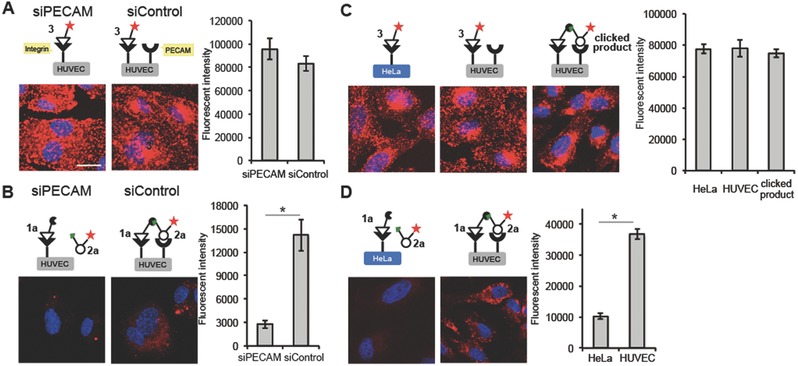
Selective imaging of HUVECs expressing both α_V_β_3_ integrin and the platelet endothelial cell adhesion molecule (PECAM). A) HUVECs, which had been transfected with siRNA against PECAM (siPECAM) or nontargeted siRNA (siControl), were treated with TAMRA‐labeled RGDyK ligand **3** (red). B) HUVECs, transfected with siPECAM or siControl, were treated with **1a** followed by **2a** (red). C) HeLa cells and HUVECs were treated with **3** (red) or with the preclicked product between **1a** and **2a** (red). D) HeLa cells and HUVECs were treated with **1a** followed by **2a** (red). After treatment with the ligands, the cells were fixed and stained with DAPI (blue). The scale bar indicates 20 µm. All data are presented as the means ± S.E. [*n* = 10 (10 000 cells × 10), Student's *t* test, *p < 0.001].

We further evaluated the method by selectively imaging HUVECs in the presence of HeLa cells. HeLa cell overexpresses the integrins but not the PECAM. Initially, the TAMRA‐labeled RGDyK 3, which has been used to image tumor cells, could not distinguish these cells (Figure 4C, left and middle). The two receptors were targeted using our method, i.e., ligands to both the integrins and PECAM were applied, and the HUVECs were successfully imaged with a fourfold fluorescence intensity contrast relative to the HeLa cell background (Figure 4D). It should be noted that the utilization of the preclicked product of a reaction between the peptide **1a** and the glycan **2a** (Figure 4C, right), did not yield better contrast than was obtained in the image collected after the addition of the TAMRA‐labeled peptide **3** (Figure 4C, left and middle). The effects of the weak glycan interaction on the preclicked molecule could be overridden by strong peptide interactions. The interactions between the molecules could be controlled only through the strongly interacting peptide components. The combination of the strong and the weak ligand/receptor interactions and the in situ click ligation on the cell surface (see Figure 4B,D) enabled cell‐selective targeting.

In molecular imaging research, selectivity is a key issue for ensuring a high image contrast. Previous studies have focused in many cases on the development of high‐affinity ligands to the surface receptors of interests. The use of a low‐affinity ligand, such as the glycan used in this study, was advantageous in that the excess labeled ligand could be washed away from the cells, whereas the ligand bound to the cell surface, even at *K*_D_ of milli molar affinity level, could be tightly anchored by a reaction with a pretargeted high‐affinity ligand on the cell surface. Therefore, nonspecific fluorescence background could be minimized. The glycan‐saturation experiments, utilization of asialoglycan **2b** as a nonspecific ligand of the target lectin, and lectin‐knockdown experiments strongly suggested the importance of applying a weak glycan/lectin interaction in our method. Note that the in situ ligation of weakly interacting ligands to a pretargeted strongly interacting ligand on the target cell was the key to obtaining a high labeling affinity and specificity. The prelinked glycan/peptide conjugate did not display the advantages of the weak glycan interaction, as the strongly interacting peptide component dominated the binding characteristics of the conjugate. The interactions of the whole molecule were then controlled only by the strong interaction.

The affinity of a single molecule of glycan toward lectin is quite low. Multivalency effects, e.g., introducing glycoclusters onto proteins, using dendrimers, liposome templates, or even microchips, are therefore used to detect lectins both in vitro and in vivo.^29^, ^30^, ^31^, ^32^, ^33^, ^34^, ^35^, ^36^ Our method uses the weak interaction of monovalent glycan to our advantage by enhancing the labeling selectivity.

The antibody‐based methods, e.g., homogeneous time resolved fluorescence (HTRF) immunoassay^37^ or proximity ligation assay,^38^ are also useful in sensitively detecting the interaction of two proteins. Especially, these methods are advantageous for high throughput screening or imaging of surface protein interactions. Our method, on the other hand, which utilizes the “strong” and the “weak” combination of two small surface ligands, would be compatible with both in vitro (cell‐based experiments described in this research) and even in vivo application (such as molecular imaging), because the low molecular‐weight ligands show favorable in vivo pharmacokinetics. In addition, various fluorescent and other labels can easily be optimized, e.g., using near‐infrared dyes or even radioactive labels. Our new method can expand the applicability of using the small ligands for the selective cell surface recognition processes, in addition to the conventional antibody‐based strategy.

Finally, it should be noted that our system efficiently bridges the integrin to PECAM on a cell surface by two cell surface ligands. The cell surface orientation and conformation of two receptors, i.e., PECAM and integrin, are dynamic, hence their ligands could react each other when receptors are close enough on the ligand binding. While crystallographic data of PECAM or detailed information of integrin/PECAM complex on cell surface are not currently available, the proximal information has been discussed based on the experimental evidences that (1) PECAM deficiency led to the loss of integrin activation,^39^ and (2) microscopic analysis of two receptors detected their colocalization on a cell surface.^24^

Thus, another attractive feature of the approach described in this paper is that this method may be used to directly analyze the relative spatial arrangements of two and possibly more receptors on a cell surface, in dynamic fashion, by using linkers of various lengths. Cell surface dynamics, as indicated by the spatial arrangements of the target proteins and, hence, the ligand‐directed signaling pathways, could be monitored or even controlled simply by treating the live cells with chemical reagents and subsequently imaging.

In conclusion, we developed a selective cell targeting strategy based on strong and weak ligand/receptor interactions and in situ click ligation on a cell surface. This method achieved a high selectivity and good imaging contrast in the presence of other background cells. We demonstrated that the target cell could be selectively visualized based on the fluorescence readout. This strategy is applicable to a variety of in vivo molecular imaging systems.

## Conflict of Interest

The authors declare no conflict of interest.

## Supporting information

Supplementary
