# Supplementary material for: In Situ Ligation of High‐ and Low‐Affinity Ligands to Cell Surface Receptors Enables Highly Selective Recognition
Source: Adv Sci (Weinh). 2017 Jul 28;4(11):1700147. doi: 10.1002/advs.201700147 (PMC5700463; doi:10.1002/advs.201700147)
Supplement: Supplementary file 1 — Supplementary [file ADVS-4-na-s001.pdf]

Copyright WILEY-VCH Verlag GmbH & Co. KGaA, 69469 Weinheim, Germany, 2017.

ADVANCED SCIENCE Open Access

Supporting Information

for *Adv. Sci.,* DOI: 10.1002/advs.201700147

In Situ Ligation of High- and Low-Affinity Ligands to Cell Surface Receptors Enables Highly Selective Recognition

*Misako Taichi, Shogo Nomura, Ikuhiko Nakase, Rie Imamaki, Yasuhiko Kizuka, Fumi Ota, Naoshi Dohmae, Shinobu Kitazume, Naoyuki Taniguchi, and Katsunori Tanaka**

Copyright WILEY-VCH Verlag GmbH & Co. KGaA, 69469 Weinheim, Germany, 2016.

Supporting Information

In-situ ligation of high- and low- affinity ligands to cell surface receptors enables highly selective recognition

*Misako Taichi, Shogo Nomura, Ikuhiko Nakase, Rie Imamaki, Yasuhiko Kizuka, Fumi Ota, Naoshi Dohmae, Shinobu Kitazume, Naoyuki Taniguchi, and Katsunori Tanaka**

General information.

Boc-miniPEG™, Boc-miniPEG-3™, and EDC・HCl were obtained from Peptide Institute, Inc. (Osaka, Japan). All other chemicals and solvents of special grade were obtained from Tokyo Chemical Industry, co., Ltd (Tokyo, Japan) or Wako Pure Chemical Industries, Ltd (Osaka, Japan), and were used without purification. HPLC was performed on Shimadzu liquid chromatograph Model LC-20AD and LC-20AP (Kyoto, Japan) with an analytical column YMC-Pack ODS (4.6 mm x 150 mm) at a flow rate of 1 mL/min, a semi-preparative column COSMOSIL 5C_{18}-AR-300 (10 mm x 250 mm) at a flow rate of 3 mL/min, and a preparative column COSMOSIL 5C_{18}-AR-300 (20 mm x 250 mm) at a flow rate of 7 mL/min. Mass spectra were recorded on a Bruker micrOTOF QIII (Rheinstetten, Germany) or Bruker MALDI-TOF MS (Rheinstetten, Germany). NMR spectra were recorded on a JEOL AL400 or ECA600 spectrometer (Tokyo, Japan).

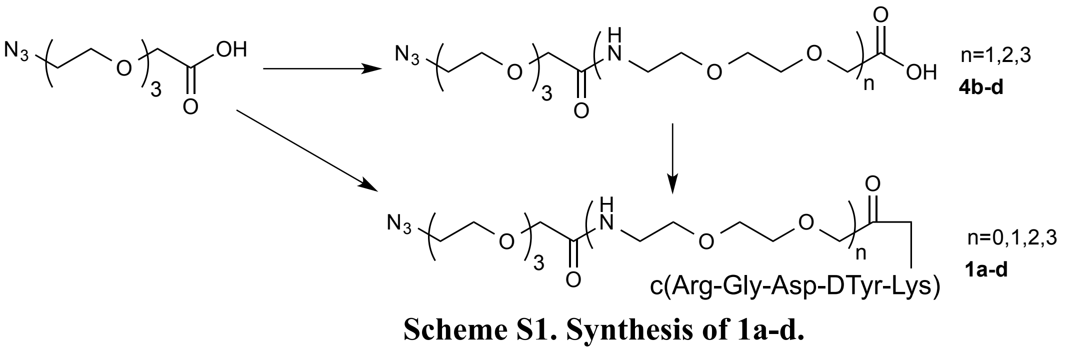

Scheme S1. Synthesis of 1a-d.

Synthesis of **4b**.

To a solution of 11-azido-3,6,9-trioxaundecanoic acid (100 mg, 0.429 mmol) in MeCN (1.0 mL) was added *N*-hydroxysuccinimide (52.0 mg, 0.450 mmol) and EDC・HCl (90.4 mg, 0.472 mmol). The reaction mixture was stirred for 3 h at room temperature, and was added to a solution of miniPEG™・TFA (119 mg, 0.429 mmol) and *N*,*N*'-diisopropylethylamine, DIEA (186 μL, 1.07 mmol) in MeCN (600 μL). The reaction mixture was stirred for 1.5 h at room temperature, quenched with AcOH (100 μL), and concentrated *in vacuo*. The resulting residue was purified by RP-HPLC using a semi-preparative column at a

flow rate of 3 mL/min with a linear gradient of MeCN/0.1%TFA (7-27% for 80 min) to obtain **4b** (120 mg, 74%). HRMS calcd for $C_{14}H_{27}N_4O_8$ ($[M+H]^+$) 379.1823, found 379.1820; ^1H NMR (400 MHz, DMSO-d_6) δ 12.61 (brs, 1H), 7.61 (dd, *J* = 5.3, 5.8 Hz, 1H), 4.00 (s, 2H), 3.86 (s, 2H), 3.49-3.61 (m, 14H), 3.43 (dd, *J* = 5.8, 6.3 Hz, 2H), 3.38 (dd, *J* = 4.4, 5.3 Hz, 2H), 3.22-3.29 (m, 4H); ^{13}C NMR (100 MHz, DMSO-d_6) δ 171.6, 169.2, 70.2, 69.9, 69.8, 69.7, 69.6, 69.5, 69.2, 68.9, 67.6, 50.0, 38.0.

Synthesis of **4c**.

To a solution of **4b** obtained above (50.0 mg, 0.132 mmol) in MeCN/H_2O (9 : 1, v/v, 2.0 mL) was added *N*-hydroxysuccinimide (16.7 mg, 0.145 mmol) and EDC・HCl (27.8 mg, 0.145 mmol). The reaction mixture was stirred for 2 h at room temperature, and was added to a solution of miniPEG^{TM}・TFA (38.5 mg, 0.139 mmol) and DIEA (57 μL, 0.33 mmol) in H_2O (500 μL). The reaction mixture was stirred for 2.5 h at room temperature, quenched with AcOH (100 μL), and concentrated *in vacuo*. The resulting residue was purified by RP-HPLC using a semi-preparative column at a flow rate of 3 mL/min with a linear gradient of MeCN/0.1%TFA (10-30% for 80 min) to obtain **4c** (67.0 mg, 87%). HRMS calcd for $C_{20}H_{38}N_5O_{11}$ ($[M+H]^+$) 524.2562, found 524.2572; ^1H NMR (400 MHz, DMSO-d_6) δ 12.51 (brs, 1H), 7.62 (dd, *J* = 5.3, 11.1 Hz, 2H), 4.00 (s, 2H), 3.87 (s, 2H), 3.86 (s, 2H), 3.49-3.61 (m, 18H), 3.41-3.45 (m, 4H), 3.39 (dd, *J* = 4.3, 5.3 Hz, 2H), 3.26 (dt, *J* = 5.8, 11.6 Hz, 4H); ^{13}C NMR (100 MHz, DMSO-d_6) δ 171.6, 169.3, 169.2, 70.2, 70.1, 69.9, 69.8, 69.7, 69.6, 69.4, 69.3, 69.2, 68.9, 67.5, 50.0, 37.9.

Synthesis of **4d**.

To a solution of **4c** obtained above (17.8 mg, 34.0 μmol) in MeCN/H_2O (9 : 1, v/v, 150 μL) was added *N*-hydroxysuccinimide (4.1 mg, 36 μmol) and EDC・HCl (7.2 mg, 37 μmol). The reaction mixture was stirred for 2.5 h at room temperature, and was added to a solution of miniPEG^{TM}・TFA (9.4 mg, 34 μmol) and DIEA (15 μL, 85 μmol) in 50% MeCN/H_2O (100 μL). The reaction mixture was stirred for 2 h at room temperature, quenched with AcOH (30 μL), and concentrated *in vacuo*. The resulting residue was purified by RP-HPLC using a semi-preparative column at a flow rate of 3 mL/min with a linear gradient of MeCN/0.1%TFA (10-30% for 80 min) to obtain **4d** (14.0 mg, 62%). HRMS calcd for $C_{26}H_{49}N_6O_{14}$ ($[M+H]^+$) 669.3301, found 669.3331; ^1H NMR (400 MHz, DMSO-d_6) δ 12.53 (brs, 1H), 7.58-7.70 (m, 3H), 4.34 (s, 2H), 3.87 (s, 2H), 3.86 (s, 4H), 3.49-3.61 (m, 22H), 3.36-3.46 (m, 8H), 3.22-3.30 (m, 6H); ^{13}C NMR (100 MHz, DMSO-d_6) δ 171.6, 169.3, 169.22, 169.19, 70.2, 70.1, 69.9, 69.8, 69.7, 69.6, 69.4, 69.2, 69.2, 68.87, 68.8, 67.5, 52.1, 49.9.

Synthesis of **1a**.

To a solution of 11-azido-3,6,9-trioxaundecanoic acid (4.5 mg, 19.3 μmol) in MeCN (200 μL) was added *N*-hydroxysuccinimide (2.3 mg, 20 μmol) and EDC・HCl (4.0 mg, 21 μmol). The reaction mixture was stirred for 2 h at room temperature, and was added to a solution of cyclo-(Arg-Gly-Asp-DTyr-Lys) (10.0 mg, 16.1 μmol) and DIEA (8.4 μL, 48 μmol) in H_2O (50 μL). The reaction mixture was stirred for 2 h at room temperature, quenched with AcOH (30 μL), and concentrated *in vacuo*. The resulting residue was purified by RP-HPLC using a preparative column at a flow rate of 7 mL/min with a linear gradient of MeCN/0.1%TFA (10-30% for 80 min) to obtain **1a** (6.8 mg, 51%). HRMS calcd for $C_{35}H_{55}N_{12}O_{12}$ ($[M+H]^+$) 835.4057, found 835.4062; ^1H NMR (400 MHz, DMSO-d_6) δ 6.99 (d, *J* = 8.2 Hz, 2H), 6.71 (d, *J* = 8.2 Hz, 2H), 4.38-4.45 (m, 1H), 4.19-4.26 (m, 1H), 4.07 (d, *J* = 15.0 Hz, 1H), 3.93 (s, 2H), 3.65-3.72 (m, 1H), 3.51-3.65 (m, 10H), 3.31-3.38 (m, 3H), 2.95-

3.13 (m, 4H), 2.68-2.91 (m, 3H), 2.55-2.64 (m, 1H), 1.66-1.79 (m, 1H), 1.20-1.68 (m, 7H), 0.73-0.90 (m, 2H); t_R: 12.0 min (flow rate of 1 mL/min with a linear gradient from 10-60% MeCN/0.1%TFA for 25 min using the analytical column).

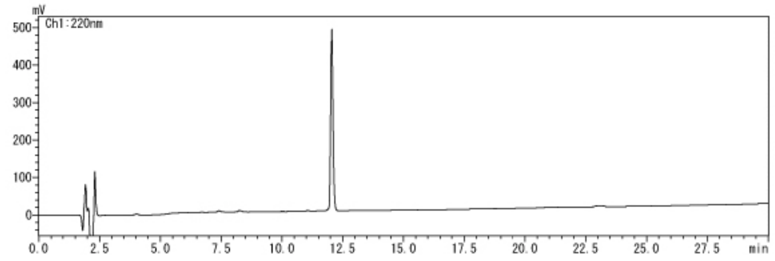

Figure S1. HPLC chromatogram of 1a.

Synthesis of **1b**.

To a solution of **4b** obtained above (10.0 mg, 26.4 μmol) in MeCN/H_2O (97 : 3, v/v, 300 μL) was added *N*-hydroxysuccinimide (3.2 mg, 28 μmol) and EDC・HCl (5.6 mg, 29 μmol). The reaction mixture was stirred for 1.5 h at room temperature, and was added to a solution of cyclo-(Arg-Gly-Asp-DTyr-Lys) (16.3 mg, 26.4 μmol) in 40% MeCN/H_2O (150 μL) and DIEA (14 μL, 79 μmol). The reaction mixture was stirred for 3 h at room temperature, quenched with AcOH (50 μL), and concentrated *in vacuo*. The resulting residue was purified by RP-HPLC using the preparative column at a flow rate of 7 mL/min with a linear gradient of MeCN/0.1%TFA (13-33% for 80 min) to obtain **1b** (9.1 mg, 35%). HRMS calcd for $C_{41}H_{66}N_{13}O_{15}$ ($[M+H]^+$) 980.4796, found 980.4825; ^1H NMR (400 MHz, DMSO-d_6) δ 6.98 (d, *J* = 7.2 Hz, 2H), 6.70 (d, *J* = 6.8 Hz, 2H), 4.38-4.45 (m, 1H), 4.19-4.26 (m, 1H), 4.07 (d, *J* = 14.5 Hz, 1H), 3.91 (s, 4H), 3.66-3.71 (m, 1H), 3.47-3.71 (m, 16H), 3.28-3.43 (m, 5H), 2.95-3.11 (m, 4H), 2.82-2.91 (m, 1H), 2.65-2.81 (m, 2H), 2.59 (dd, *J* = 6.3, 16.4 Hz, 1H), 1.65-1.77 (m, 1H), 1.44-1.58 (m, 2H), 1.29-1.44 (m, 3H), 1.17-1.29 (m, 2H), 0.72-0.90 (m, 2H); t_R: 12.4 min (flow rate of 1 mL/min with a linear gradient from 10 to 60% MeCN/0.1%TFA for 25 min using the analytical column).

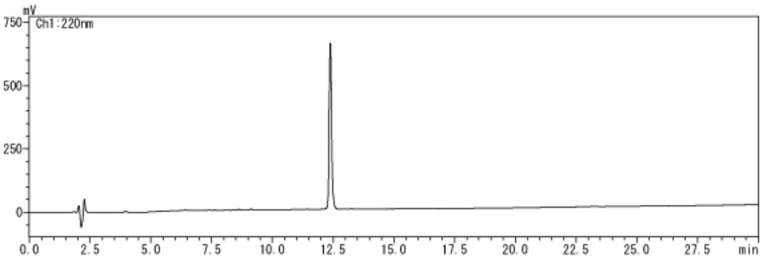

Figure S2. HPLC chromatogram of 1b.

Synthesis of **1c**.

To a solution of **4c** obtained above (5.5 mg, 11 μmol) in MeCN/H_2O (9 : 1, v/v, 150 μL) was added *N*-hydroxysuccinimide (1.3 mg, 11 μmol) and EDC・HCl (2.2 mg, 11 μmol). The reaction mixture was stirred for 2 h at room temperature, and was added to cyclo-(Arg-Gly-Asp-DTyr-Lys) (5.0 mg, 8.1 μmol) and DIEA (4.2 μL, 24 μmol). The reaction mixture was stirred for 2 h at room temperature, quenched with AcOH (20 μL), and diluted with H_2O (150 μL). The mixture was purified by RP-HPLC using the semi-preparative column at a flow rate of 3 mL/min with a linear gradient of MeCN/0.1%TFA (13-33% for 80 min) to obtain **1c** (2.9 mg, 32%). HRMS calcd for $C_{47}H_{77}N_{14}O_{18}$ ($[M+H]^+$) 1125.5535, found 1125.5579; ^1H NMR (400 MHz, DMSO-d_6) δ 6.98 (d, *J* = 8.2 Hz, 2H), 6.70 (d, *J* = 8.2 Hz, 2H), 4.38-4.45

(m, 1H), 4.19-4.25 (m, 1H), 4.07 (d, *J* = 15.0 Hz, 1H), 3.87-3.95 (m, 6H), 3.65-3.72 (m, 1H), 3.47-3.65 (m, 22H), 3.28-3.42 (m, 7H), 2.94-3.11 (m, 4H), 2.67-2.90 (m, 3H), 2.56-2.64 (m, 1H), 1.65-1.77 (m, 1H), 1.18-1.57 (m, 7H), 0.73-0.90 (m, 2H) t_R: 12.6 min (flow rate of 1 mL/min with a linear gradient from 10 to 60% MeCN/0.1%TFA for 25 min using the analytical column).

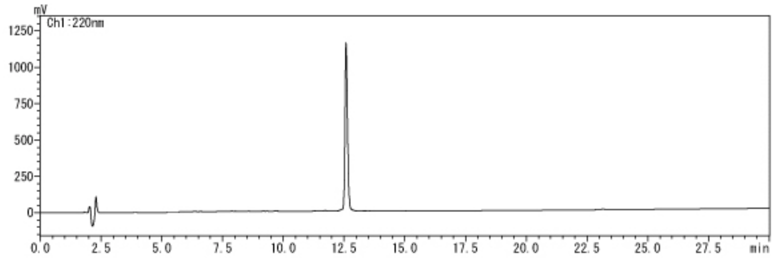

Figure S3. HPLC chromatogram of 1c.

Synthesis of **1d**.

To a solution of **4d** (10.0 mg, 15.0 μmol) in MeCN/H_2O (9 : 1, v/v, 240 μL) was added *N*-hydroxysuccinimide (1.8 mg, 16 μmol) and EDC・HCl (3.2 mg, 17 μmol). The reaction mixture was stirred for 1.5 h at room temperature, and was added to cyclo-(Arg-Gly-Asp-DTyr-Lys) (9.3 mg, 15 μmol) and DIEA (7.8 μL, 45 μmol). The reaction mixture was stirred for 2 h at room temperature, quenched with AcOH (20 μL), and concentrated *in vacuo*. The resultant residue was purified by RP-HPLC using the preparative column at a flow rate of 7 mL/min with a linear gradient of MeCN/0.1%TFA (13-33% for 80 min) to obtain **1d** (9.0 mg, 47%). HRMS calcd for $C_{53}H_{88}N_{15}O_{21}$ ($[M+H]^+$) 1270.6274, found 1270.6307; ^1H NMR (400 MHz, DMSO-*d*$_6$) δ 6.89 (d, J = 8.2 Hz, 2H), 6.70 (d, J = 8.2 Hz, 2H), 4.38-4.46 (m, 1H), 4.19-4.26 (m, 1H), 4.07 (d, J = 15.0 Hz, 1H), 3.89-3.97 (m, 8H), 3.66-3.72 (m, 1H), 3.48-3.64 (m, 28H), 3.29-3.38 (m, 9H), 2.95-3.12 (m, 4H), 2.66-2.91 (m, 3H), 2.55-2.66 (m, 1H), 1.63-1.78 (m, 1H), 1.15-1.60 (m, 7H), 1.72-1.91 (m, 2H); t_R: 12.9 min (flow rate of 1 mL/min with a linear gradient from 10 to 60% MeCN/0.1%TFA for 25 min using the analytical column).

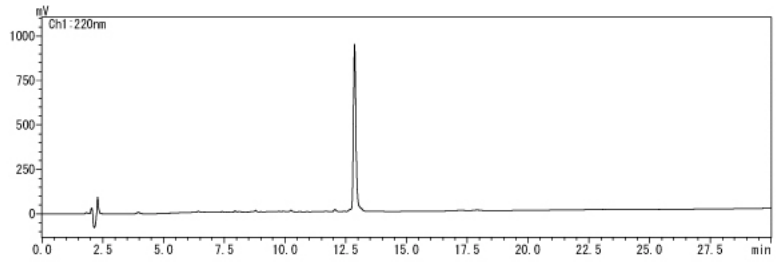

Figure S4. HPLC chromatogram of 1d.

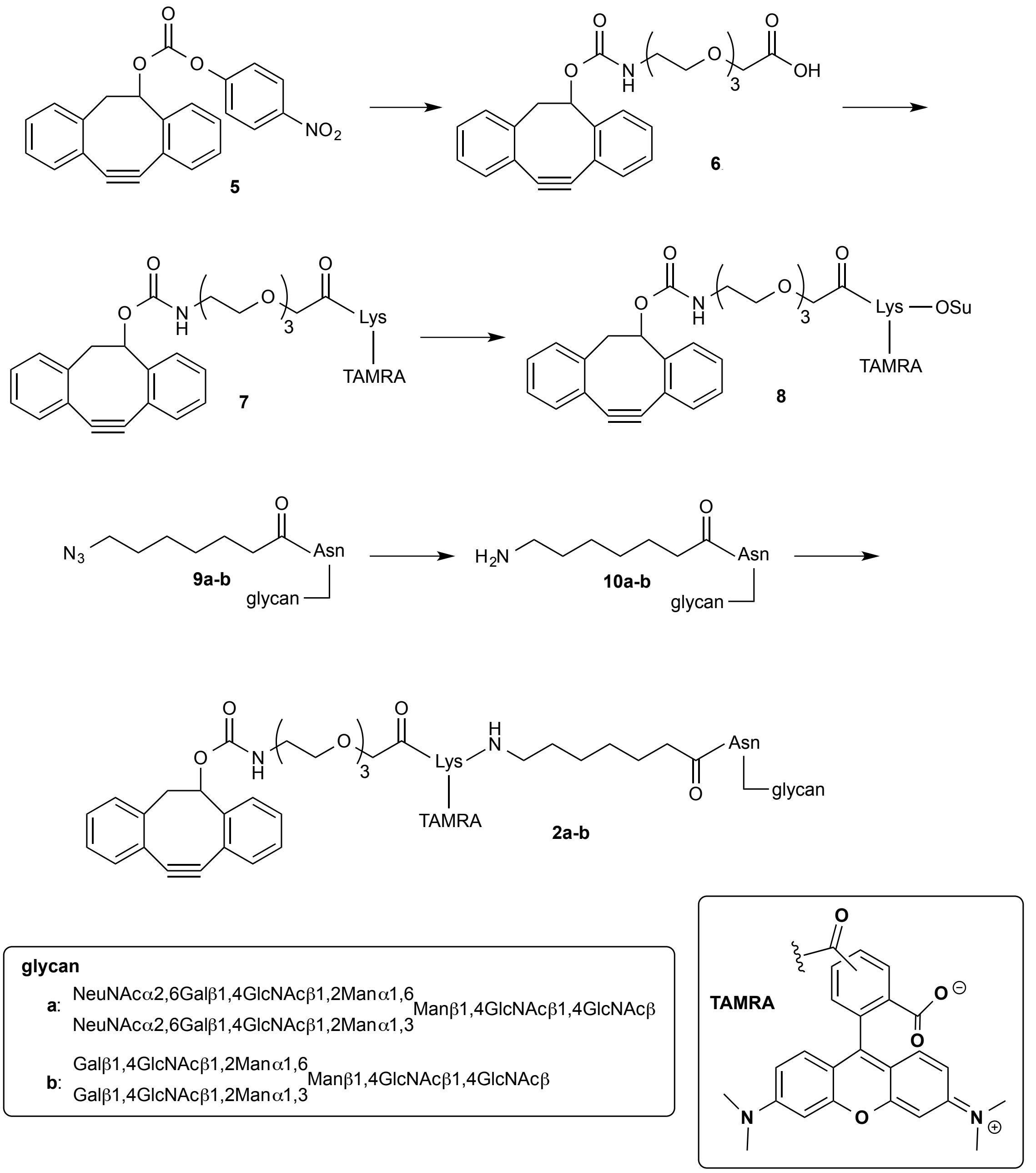

Scheme S2. Synthesis of 2a and b.

Synthesis of **6**.

To a solution of **5**[1] (32.0 mg, 83.0 μmol) in MeCN (1.0 mL) was added miniPEG-3™ • TFA (40.0 mg, 0.125 mmol) and DIEA (51 μL, 0.29 mmol). The reaction mixture was stirred for 3 h at room temperature, quenched with AcOH (200 μL), and concentrated *in vacuo*. The resultant residue was purified by RP-HPLC using the semi-preparative column at a flow rate of 3 mL/min with a linear gradient of MeCN/0.1%TFA (35-60% for 80 min) to obtain **6** (37.6 mg, quant). HRMS calcd for $C_{25}H_{28}NO_7$ ($[M+H]^+$) 454.1860, found 454.1873; ^{1}H NMR (400 MHz, DMSO-d_6) δ 12.52 (brs, 1H), 7.28-7.66 (m, 9H), 5.28 (s, 1H), 4.00 (s, 2H), 3.35-3.62 (m, 10H), 3.10-3.20 (m, 3H), 2.75 (dd, J = 3.9, 15.0 Hz, 1H); ^{13}C NMR (100 MHz, DMSO-d_6) δ 171.6, 155.3, 152.4, 150.9, 130.2, 128.4, 127.4, 127.3, 126.1, 125.8, 123.8, 122.9, 120.3, 112.6, 75.3, 69.8, 69.7, 69.6, 69.6, 69.5, 69.0, 67.5, 45.5.

Preparation of lysine labeled by 5(6)-carboxytetramethylrhodamine (TAMRA) at the ε-amino group (Lys(TAMRA)・TFA salt).

To a solution of Boc-Lys (2.5 mg, 10 μmol) in DMF/H_2O (250/120 μL) was added 5(6)-carboxytetramethylrhodamine succinimidyl ester (as two regioisomers, purchased from Life technologies, C1171, 5.0 mg, 9.5 μmol) and DIEA (4.9 μL, 28 μmol). The reaction mixture was stirred for 3 h at room temperature, quenched by AcOH (50 μL), and purified by RP-HPLC using the semi-preparative column at a flow rate of 3 mL/min with a linear gradient of MeCN/0.1%TFA (25-50% for 80 min) to obtain Boc-Lys(TAMRA) (5.7 mg). Boc-Lys(TAMRA) thus obtained was dissolved in TFA (200 μL) and the reaction mixture was stirred for 10 min at room temperature. After removal of excess TFA with N_2 gas, the resultant residue was dissolved in H_2O, and lyophilized to obtain Lys(TAMRA)・TFA (5.4 mg, 85%) as a mixture of TAMRA regioisomers. HRMS calcd for $C_{31}H_{35}N_4O_6$ ($[M+H]^+$) 559.2551, found 559.2575 for the isomer t_R: 13.2 min, 559.2569 for the isomer t_R: 14.8 min (flow rate of 1 mL/min with a linear gradient from 10 to 60% MeCN/0.1%TFA for 25 min using the analytical column). ^{1}H NMR (400 MHz, D_2O, for the isomer t_R: 13.2 min) δ 8.02 (d, *J* = 8.2 Hz, 1H), 7.91 (d, *J* = 8.2 Hz, 1H), 7.58, (s, 1H), 6.97 (d, *J* = 13.18 Hz, 1H), 6.70 (d, *J* = 9.66 Hz, 1H), 6.47 (s, 2H), 3.66-3.72 (m, 1H), 3.26-3.29 (m, 2H), 3.00 (s, 12H), 1.71-1.82 (m, 2H), 1.48-1.58 (m, 2H), 1.26-1.44 (m, 2H). (400 MHz, D_2O, for the isomer t_R: 14.8 min) δ 8.33 (s, 1H), 7.98 (d, *J* = 4.8 Hz, 1H), 7.43 (d, *J* = 6.8 Hz, 1H), 7.00-7.18 (m, 2H), 6.68-6.77 (m, 2H), 6.28-6.38 (m, 2H), 3.73-3.81 (m, 1H), 3.34-3.43 (m, 2H), 2.99 (s, 12H), 1.79-1.94 (m, 2H), 1.57-1.69 (m, 2H), 1.33-1.54 (m, 2H).

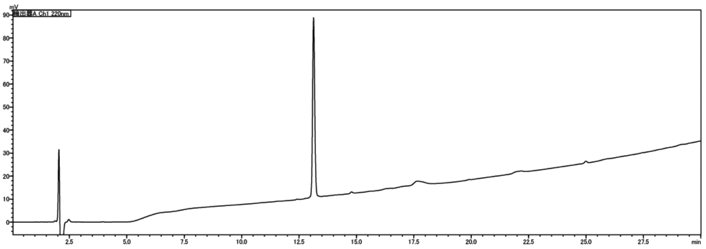

Figure S5. HPLC chromatogram of Lys(TAMRA) (t_R: 13.2 min).

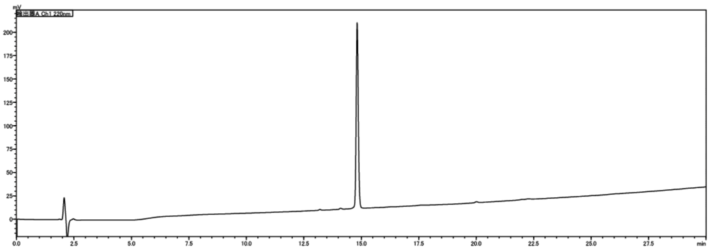

Figure S6. HPLC chromatogram of Lys(TAMRA) (t_R:14.8 min).

Synthesis of **7**.

To a solution of **6** (7.0 mg, 15 μmol) in 90% MeCN (200 μL) was added *N*-hydroxysuccinimide (1.9 mg, 17 μmol) and EDC・HCl (3.2 mg, 17 μmol). The reaction mixture was stirred for 2 h at room temperature, and was added to Lys(TAMRA) prepared above (8.6 mg, 13 μmol) and DIEA (8.9 μL, 64 μmol). The reaction mixture was stirred for 1 h at room temperature, quenched with AcOH (30 μL), and concentrated *in vacuo*. The residue was purified by RP-HPLC using the semi-preparative column at a flow rate of 3 mL/min with a linear gradient of MeCN/0.1%TFA (30-50% for 80 min) to obtain **7** (14 mg, quant) as a

mixture of regioisomers. HRMS calcd for $C_{56}H_{60}N_5O_{12}$ ($[M+H]^+$) 994.4233, found 994.4254 for the isomer t_R: 19.8 min, 994.4184 for the isomer t_R: 21.1 min (flow rate of 1 mL/min with a linear gradient from 20 to 70% MeCN/0.1%TFA for 25 min using the analytical column). ^{1}H NMR spectrum is shown below.

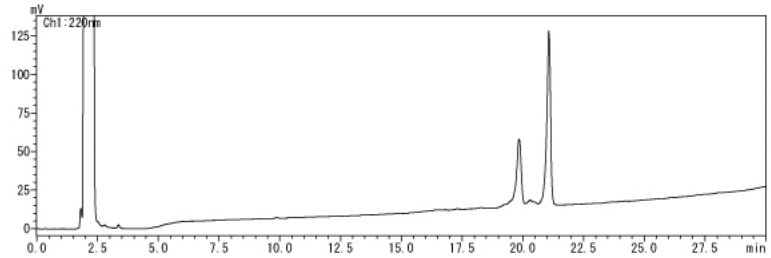

Figure S7. HPLC chromatogram of 7.

Synthesis of **8**.

To a solution of **7** obtained above (8.9 mg, 9.0 μmol) in DMF (200 μL) was added *N*-hydroxysuccinimide (2.1 mg, 18 μmol) and EDC・HCl (10.3 mg, 53.7 μmol). The solution was stirred for 1.5 h at room temperature, and the mixture was directly purified by RP-HPLC using the semi-preparative column at a flow rate of 3 mL/min with a linear gradient of MeCN/0.1%TFA (30-60% for 80 min) to obtain **8** (6.7 mg, 68%) as a mixture of regioisomers. HRMS calcd for $C_{60}H_{63}N_6O_{14}$ ($[M+H]^+$) 1091.4397, found 1091.4356 for the isomer t_R: 15.7 min, 1091.4424 for the isomer t_R: 16.7 min (flow rate of 1 mL/min with a linear gradient from 30 to 80% MeCN/0.1%TFA for 25 min using the analytical column).

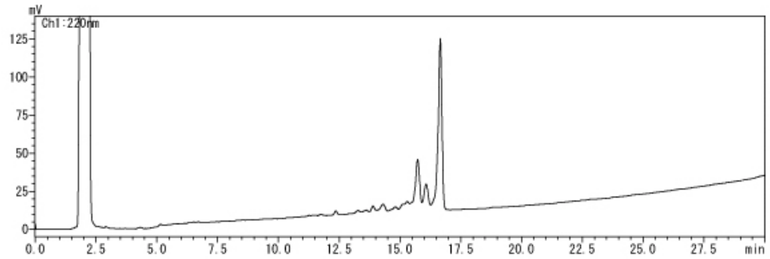

Figure S8. HPLC chromatogram of 8.

Synthesis of **2a**.

To a solution of **9a**[2] (3.0 mg, 1.2 μmol) in MeOH/H_2O (2 : 1, v/v, 1 mL) was added 10% palladium on carbon (wet with 55% H_2O, 320 μg). After the reaction mixture was stirred under H_2 atmosphere for 4.5 h, the catalyst was removed by filtration, and concentrated *in vacuo*. The residue was dissolved in H_2O, and lyophilized to obtain the amine **10a** (3.4 mg, quant), which was used for the reaction with **8** without further purification. HRMS calcd for $C_{95}H_{159}N_9O_{65}$ ($[M+2H]^{2+}$) 1232.9707, found 1232.9750.

To a solution of **8** (1.1 mg, 970 nmol) in DMF (50 μL) was added a solution of **10a** (1.2 mg, 480 nmol) in 0.1 M sodium phosphate buffer (pH 7.0, 100 μL). The reaction mixture was stirred for 25 h at room temperature, quenched with 50% AcOH, and purified by RP-HPLC using the semi-preparative column at a flow rate of 3 mL/min with a linear gradient of MeCN/0.1%TFA (30-60% for 80 min) to obtain **2a** (1.1 mg, 66%) as a mixture of TAMRA regioisomers. HRMS calcd for $C_{151}H_{217}N_{14}O_{76}$ ($[M+2H]^{2+}$) 1720.6734, found 1720.6808 for the isomer t_R: 16.2 min, 1720.6786 for the isomer t_R: 17.1 min; ^{1}H NMR spectrum is shown below. t_R: 17.1 min, isomer: 16.2 min (flow rate of 1 mL/min with a linear gradient from 20 to 70% MeCN/0.1%TFA for 25 min using the analytical column).

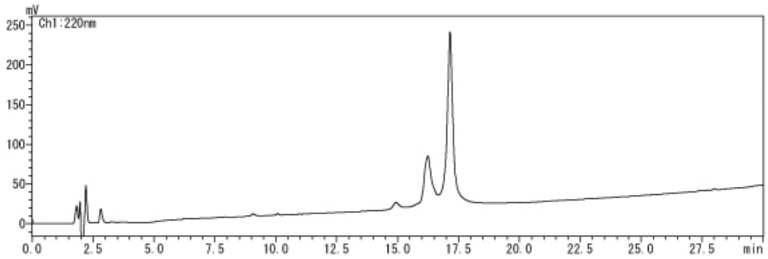

Figure S9. HPLC chromatogram of 2a.

Synthesis of **2b**.

To a solution of **9b**[2] (3.2 mg, 1.68 μmol) in MeOH/H_2O (2 : 1, v/v, 500 μL) was added 10% palladium on carbon (wet with 55% H_2O, 320 μg). After the reaction mixture was stirred under H_2 atmosphere for 4 h, the catalyst was removed by filtration, and concentrated *in vacuo*. The residue was dissolved with H_2O, and lyophilized to obtain the corresponding amine **10b** (3.0 mg, 95%), which was used for the reaction with **8** without further purification. MALDI-TOF MS: calcd for $C_{73}H_{122}N_7O_{49}$ ([M-H]$^-$) 1880.73, found 1880.57.

To a solution of **8** (870 μg, 800 nmol) in DMF (40 μL) was added a solution of **10b** obtained above (750 μg, 400 nmol) in 0.1 M sodium phosphate buffer (pH 7.0, 80 μL). The reaction mixture was stirred for 24 h at room temperature, quenched with 50% AcOH and purified by RP-HPLC using the semi-preparative column at a flow rate of 3 mL/min with a linear gradient of MeCN/0.1%TFA (30-60% for 80 min) to obtain **2b** (600 μg, 53%) as a mixture of TAMRA regioisomers. HRMS calcd for $C_{129}H_{180}N_{12}O_{60}$ ([M+2H]$^{2+}$) 1429.5780, found 1429.5770 for the isomer t_R: 16.2 min, 1429.5800 for the isomer t_R: 17.3 min; ^{1}H NMR spectrum is shown below. ;t_R: 17.3 min, isomer: 16.2 min (flow rate of 1 mL/min with a linear gradient from 20 to 70% MeCN/0.1%TFA for 25 min using the analytical column).

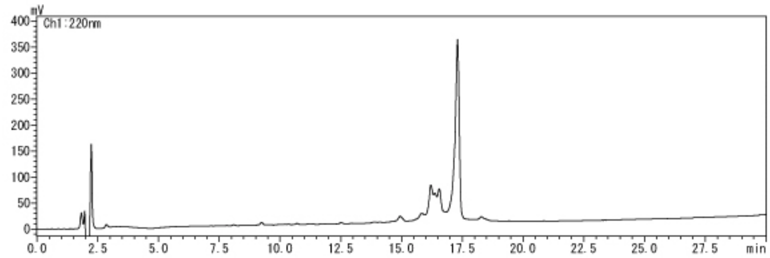

Figure S10. HPLC chromatogram of 2b.

Synthesis of cyclo-[Arg-Gly-Asp-DTyr-Lys(TAMRA)] (**3**).

To a solution of cyclo-(Arg-Gly-Asp-DTyr-Lys) (1.2 mg, 1.9 μmol) in DMF (50 μL) was added 5(6)-carboxytetramethylrhodamine-OSu (1.0 mg, 2.0 μmol) and DIEA (1.3 μL, 7.5 μmol). The mixture was stirred for 3.5 h at room temperature, quenched with AcOH (10 μL), and purified by RP-HPLC using the semi-preparative column at a flow rate of 3 mL/min with a linear gradient of MeCN/0.1%TFA (17-37% for 80 min) to obtain **3** (2.1 mg, quant) as a mixture of the TAMRA regioisomers. HRMS calcd for $C_{52}H_{62}N_{11}O_{12}$ ([M+H]$^+$) 1032.4574, found 1032.4600 for isomer t_R: 14.3 min, 1032.4609 for isomer t_R: 16.0 min (flow rate of 1 mL/min with a linear gradient from 10 to 60% MeCN/0.1%TFA for 25 min using the analytical column).

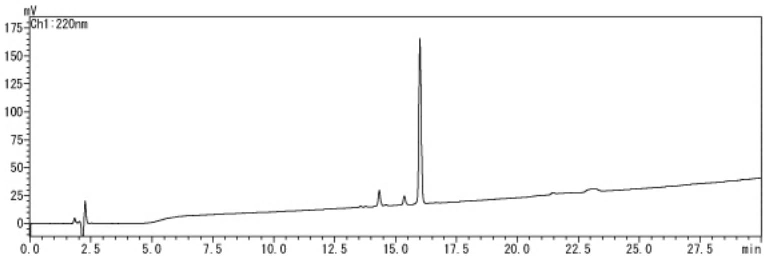

Figure S11. HPLC chromatogram of cyclo-[Arg-Gly-Asp-DTyr-Lys(TAMRA)] (3).

Preparation of authentic sample of "clicked" product between **1a** and **2a**.

To a solution of **2a** (0.77 mg, 0.33 μmol) in PBS (50 μL) was added **1a** (0.22 mg, 0.26 μmol. The reaction mixture was stirred for 1.5 h at room temperature, and purified by RP-HPLC using the semi-preparative column at a flow rate of 3 mL/min with a linear gradient of MeCN/0.1%TFA (20-50% for 80 min) to obtain the clicked product (0.4 mg for the isomer t_R: 17.8 min, 0.6 mg for the isomer t_R: 17.2 min). HRMS calcd for $C_{186}H_{268}N_{26}O_{88}$ ($[M+3H]^{3+}$) 1425.5765, found 1425.5845 for the isomer t_R: 17.8 min, 1425.5831 for the isomer t_R: 17.2 min.

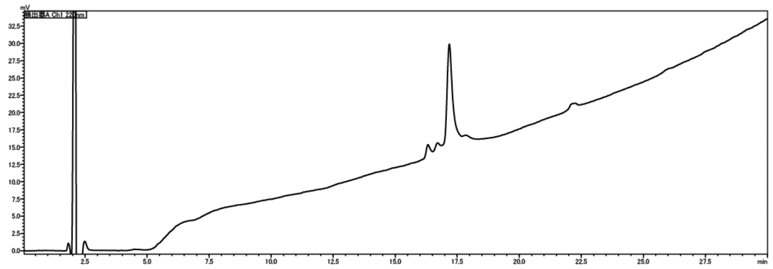

Figure S12. HPLC chromatogram of the clicked product, 1a+2a (t_R: 17.2 min).

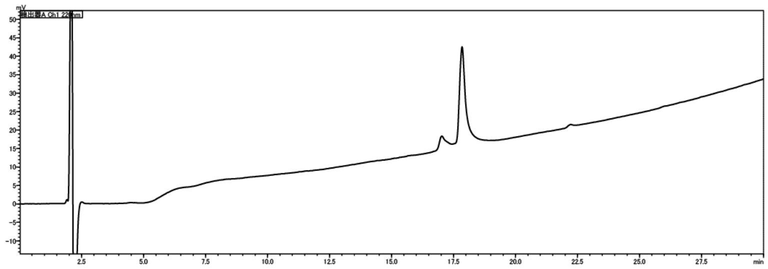

Figure S13. HPLC chromatogram of the clicked product , 1a+2a (t_R: 17.8 min).

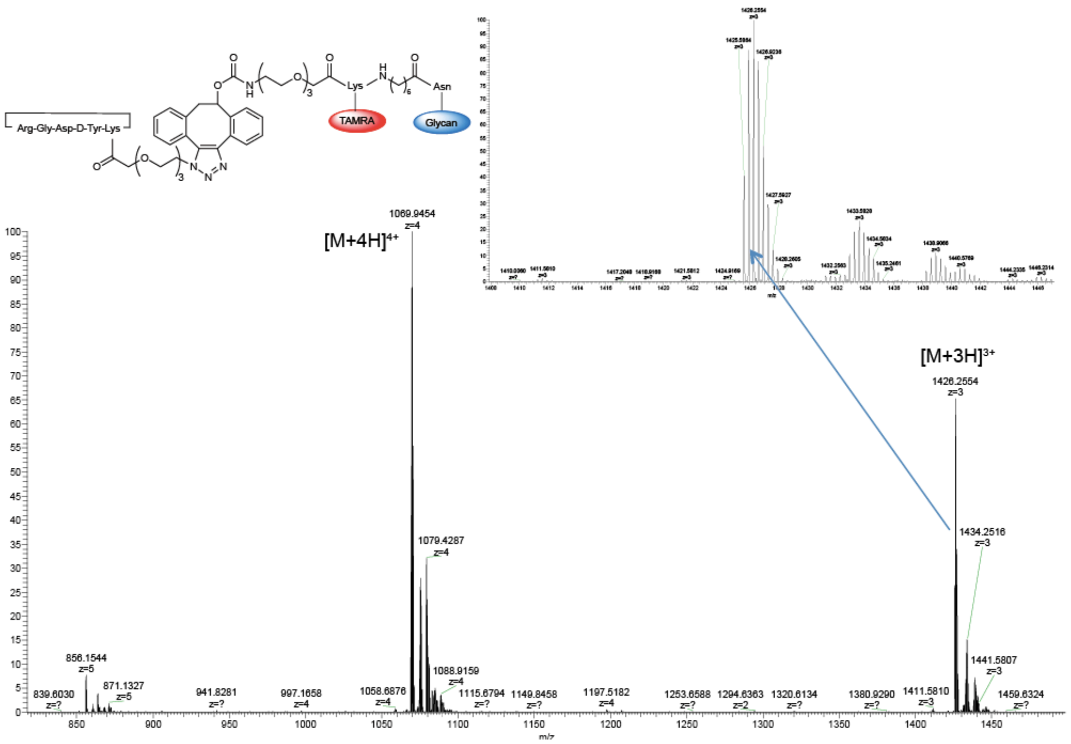

Figure S14. Mass spectrum of the clicked product, 1a+2a, as a mixture of the isomers. HRMS calculated for $C_{186}H_{268}N_{26}O_{88}$ ($[M+3H]^{3+}$]) 1425.5765, found 1425.5764.

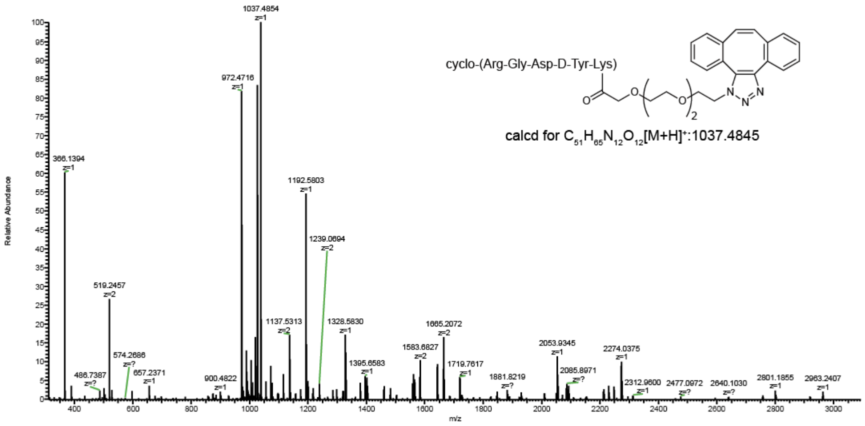

Figure S15. LC-MS/MS spectrum of the precursor ion at 1425.58 $[M+3H]^{3+}$ of the clicked product, 1a+2a). The fragment ion corresponding to the elimination product, calculated for $C_{51}H_{65}N_{12}O_{12}$ $[M+H]^{+}$: 1037.4845, found 1037.4854.

LC-MS/MS analysis of clicked products (**1a**+**2a**) from cell surface

HUVECs, which were treated with **1a** and **2a** according to the established procedure, were washed with PBS twice, and treated with KCl-HCl buffer (pH 2.0, 1.0 mL) on ice, three times for 1 min to dissociate the clicked products from the cell surface. The collected KCl-HCl buffer solution was combined and centrifuged. The supernatant was then lyophilized for MS analysis. The sample was subjected to nano-liquid chromatography-mass spectrometry. The clicked product were purified using nano ESI spray column (100 mm length ×75 μm inside diameter, 3 μm, NTCC analytical column C18, Nikkyo Technos Co., Ltd., Tokyo, Japan) that was equilibrated with A buffer (0.1% aqueous formic acid) and eluted with a linear gradient of 35% buffer B (0.1% formic acid in 100% acetonitrile) over 10 min at a flow

rate of 300 nl/min (Easy nLC, Thermo Fisher Scientific). The mass spectrometer (Q-Exactive, Thermo Fisher Scientific) was operated in the positive-ion mode, and the MS/MS spectra were acquired in a data-dependent TOP10 method or a targeted MS/MS (*m*/*z* = 1425.58) method.

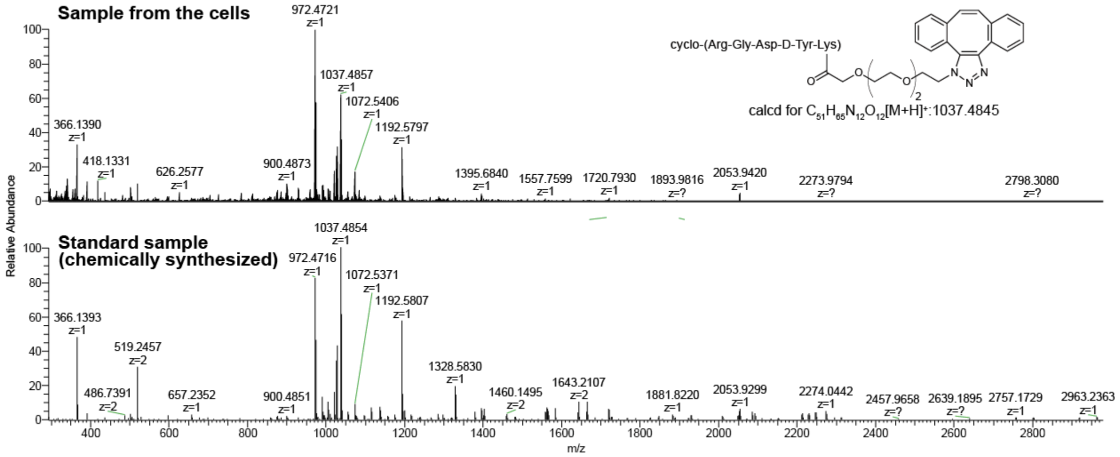

Figure S16. LC-MS/MS spectra of the precursor ion at 1425.58 $[M+3H]^{3+}$ from the clicked product, 1a+2a, isolated from HUVECs surface after applying pre-targeting method (upper) and the standard sample (lower). The fragment ion calculated for $C_{51}H_{65}N_{12}O_{12}$ $[M+H]^{+}$: 1037.4845, found 1037.4857 (upper), 1037.4854 (lower).

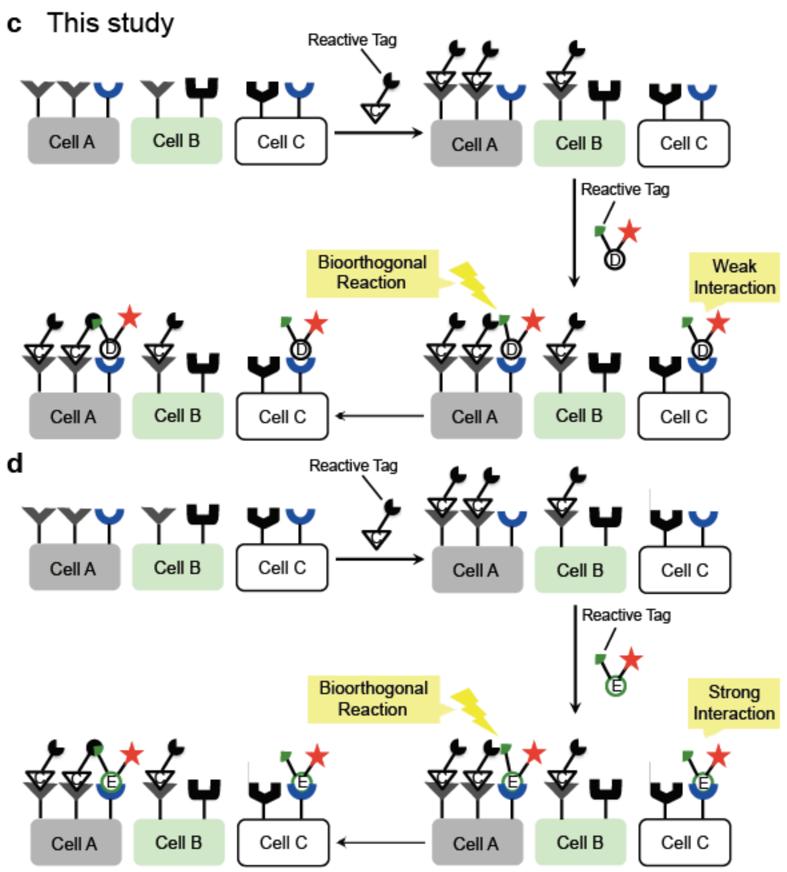

Figure S17. Importance of using low-affinity ligand of the surface receptor on the target cell A for highly selective imaging. In comparison with the Figure 1b using the low-affinity ligand "D", when applying the high-affinity ligand, e.g., ligand "E", could bind and stick to the other cells (Figure S17d), e.g., "cell C" expressing this receptor, so that the imaging selectivity is reduced.

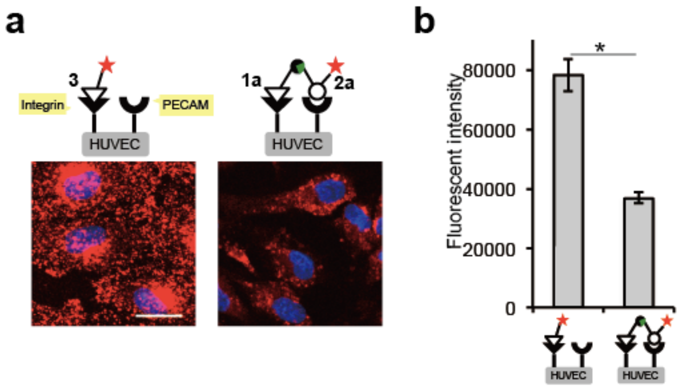

Figure S18. Evaluation of SPAAC Efficiency: Fluorescence images of HUVECs simply treated with 3 and by new pre-targeted strategy. a, HUVECs were labeled with **3** alone (red) or with the RGDyK peptide **1a** followed by **2a**, fixed, stained with DAPI, and observed by confocal microscope under the identical conditions described in Figure 3. The scale bar indicates 20 μm. **b**, Comparison of the fluorescent intensities. Data are presented as the means ± S.E. [n = 10 (10,000 cells x 10), Student's t test, *$p < 0.0001$].

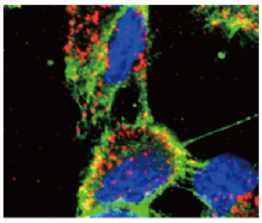

Figure S19. HUVECs doubly stained with TAMRA-labeled RGDyK peptide 3 anti-PECAM antibody. HUVECs were treated with TAMRA-labeled RGDyK peptide **3** (*red*) for 15 min at rt, then fixed, and stained with anti-PECAM (*green*) and DAPI (*blue*). The 57% of cell surface fluorescence was detected out of all fluorescence on a whole cell.

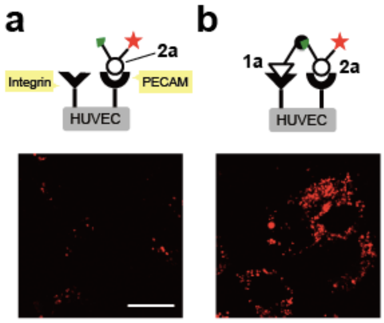

Figure S20. Live cell imaging of HUVECs using both 1a and 2a and SPAAC reaction. HUVECs were labeled using the following ligand combinations: **a**, the glycan ligand **2a** alone (*red*); **b**, the RGDyK peptide **1a** followed by **2a**. After treatment with the ligands, the cells were directly imaged by confocal microscope. The scale bar indicates 20 μm.

Flow cytometer.

HUVECs (1.2×10^5 cells, 1 mL) were plated onto a 24-well microplate (Iwaki) and incubated in the EBM^{TM}-2 cell culture medium for 24 h at 37°C under 5% CO_2. After complete adhesion, the cells were washed with the cell culture medium and treated with the cyclic RGD peptide **1a** (50 μM, 600 μl/well) for 15 min at room temperature (25°C) before washing with the EBM^{TM}-2 cell culture medium (triple washing, 200 μl). The cells were treated with the TAMRA-glycan derivative **2a** (50 μM, 600 μl/well) for 30 min at 4°C. After washing with PBS (triple washing, 200 μl), the cells were treated with 2 mM EDTA (200 μl/well) at 37°C for 10 min and then centrifuged at 3,000 rpm (800 × *g*) for 5 min at 4°C. After removal of the supernatant, the cells were washed with PBS (400 μl) and centrifuged at 3,000 rpm for 5 min at 4°C. This washing cycle was repeated, and the cells were suspended in PBS (400 μl) and subjected to fluorescence analysis with a guava easyCyte (Merck Millipore) flow cytometer using 488 nm laser excitation and a 583 nm emission filter. Live cells (10,000 cells/sample) for the detection of cellular fluorescence intensity were quantified based on forward-scattering and side-scattering analyses.

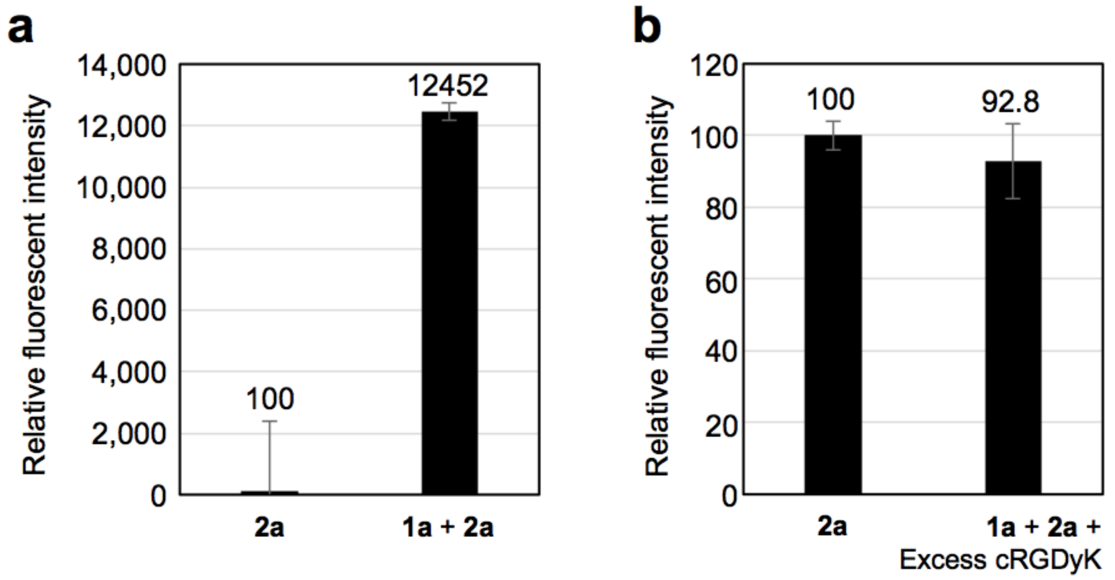

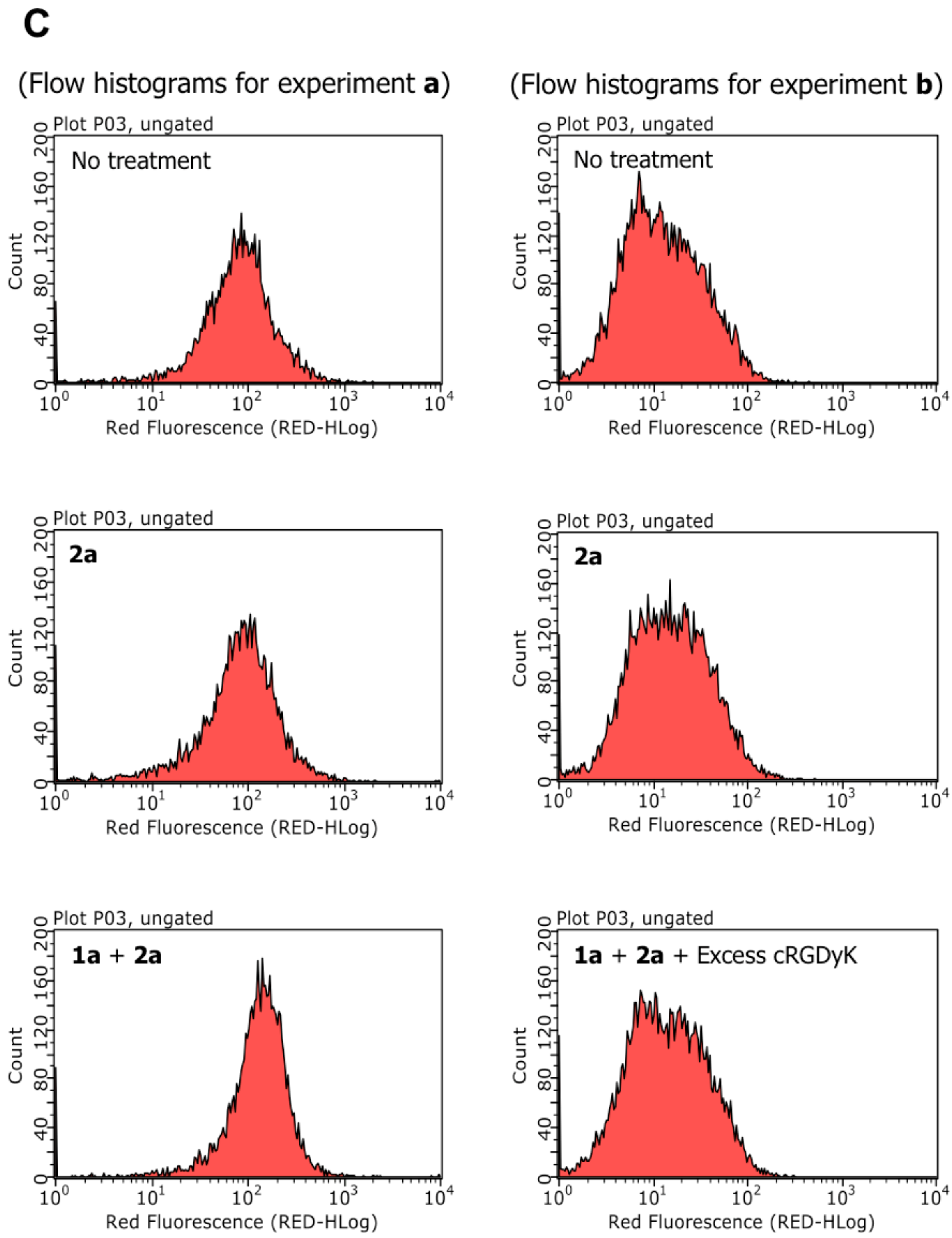

Figure S21. a, Flow cytometer analysis of HUVECs treated with TAMRA-labeled glycan **2a** (50 μM, 30 min) with or without pretreatment of cyclic RGDyK ligand **1a** (50 μM, 15 min). Experiments were performed without cell fixation as described above. **b**, Addition of cyclic RGDyK peptide without an azide moiety (250 μM) prevented the binding of TAMRA-labeled glycan **2a** in the pre-targeting experimental conditions, analyzed by flow cytometer. **c**, Flow histogram data. The data are expressed as the mean (±SD) of three experiments [n = 3 (living 10,000 cells x 3)].

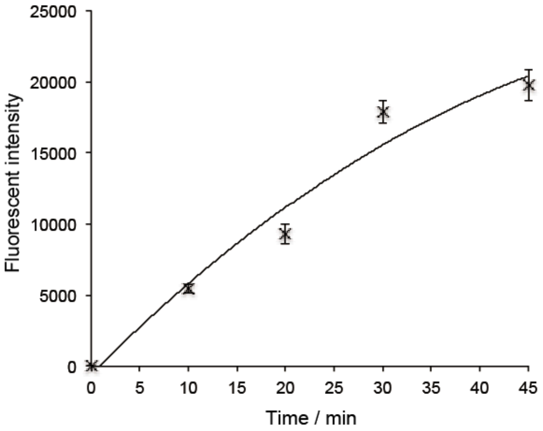

Figure S22. Time-dependent fluorescence intensity on HUVECs during cell surface SPAAC reaction between 1a and 2a. Pre-targeted RGDyK peptide **1a** on HUVECs was treated with the glycan ligand **2a** at 4 °C. Fluorescent intensities were measured by confocal microscopy and plotted at indicated time points. Fluorescence increase was measured until 45 min, since after 60 min, the shape of the ligands-treated cells gradually distorted presumably due to the cytotoxicity. Error bars represent S.E. Time-dependent fluorescence increase by the reaction with cell impermeable TAMRA-sialoglycan probe **2a** hence ensures the SPAAC reaction on cell surface.

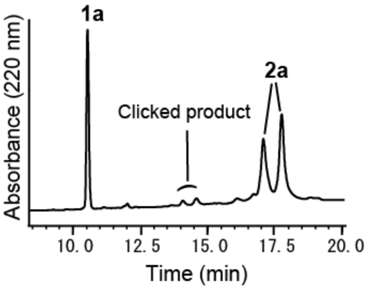

Figure S23. HPLC profile of SPAAC reaction between 1a and 2a after 30 min at 4 °C in flask.

The RGD peptide **1a** and the glycan ligand **2a** were mixed at a final concentration of 50 μM each. The reaction mixture was allowed to stand at 4 °C for 30 min, and monitored by RP-HPLC. (flow rate of 1 mL/min with a linear gradient from 10 to 80% MeCN/0.1%TFA for 25 min using the analytical column).

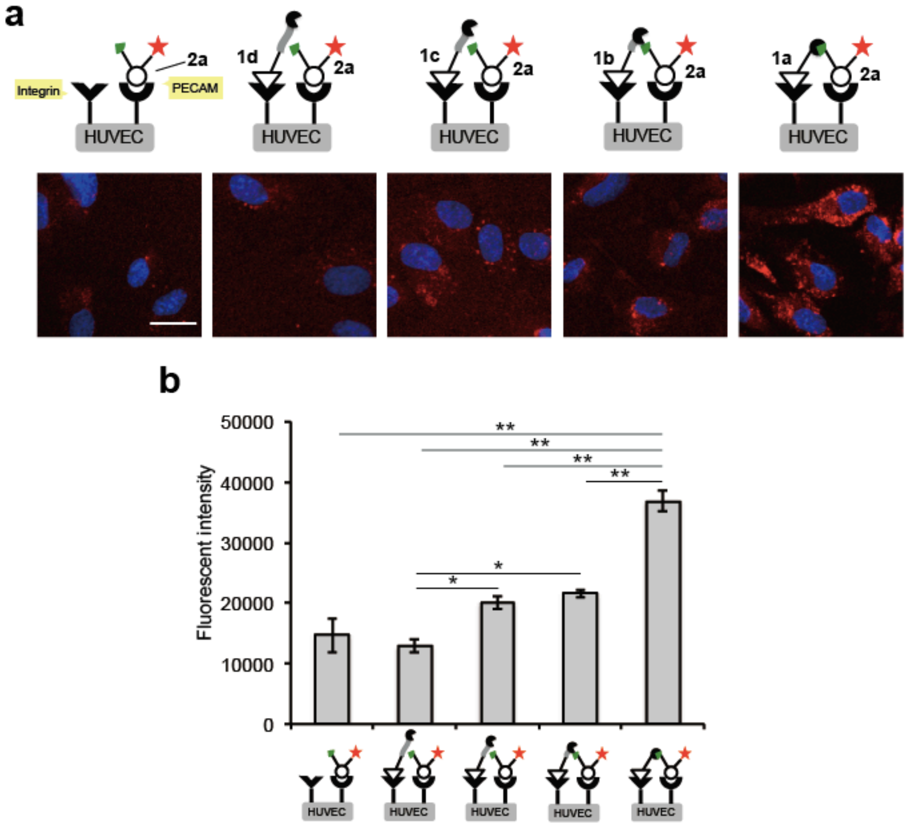

Figure S24. Effects of linker length in 1a-1d for SPAAC reaction on HUVECs. **a**, HUVECs were labeled using the ligand combination of the RGDyK peptides **1a**-**1d** with the glycan **2a** (*red*), fixed, stained with DAPI (*blue*), and observed by confocal microscopy under the identical conditions described in Figure 3. The scale bar indicates 20 μm, **b**, Comparison of the fluorescent intensities measured in Figure S23a. Data are presented as the means ± S.E. [n = 10 (10,000 cells x 10), one way ANOVA post hoc Tukey–Kramer's test, *$p<0.01$, **$p<0.05$].

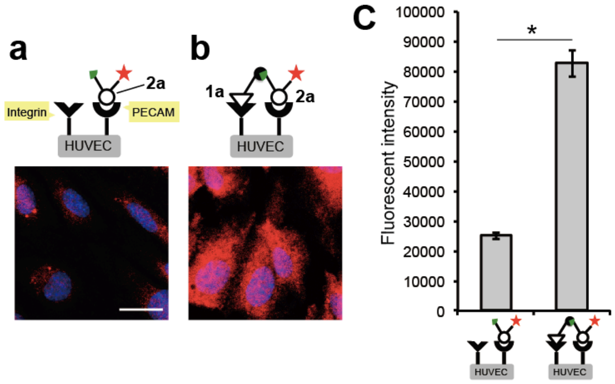

Figure S25. Imaging of HUVECs using both 1a and 2a and SPAAC reaction under cell fixing conditions. a, HUVECs were fixed with 4% paraformaldehyde (PFA) for 30 min at rt, and then labeled with the glycan ligand **2a** alone (*red*). **b**, HUVECs were treated with the RGDyK peptide **1a**, then fixed with PFA followed by treated with **2a**. After treatment with the ligands, the cells were stained with DAPI (*blue*). The scale bar indicates 20 μm. **c**, Comparison of the fluorescent intensities. Data are presented as the means ±S.E. [n = 10 (10,000 cells x 10), Student's t test, *$p < 0.0001$].

siRNA transfection experiment.

HUVECs were seeded onto 8-well chamber slides coated with type I collagen (2 × 10^4 cells/well). After siRNA transfection followed by treatment with the probes **1a** and **2a**, the cells were fixed with 4% paraformaldehyde and blocked with 5% goat serum in PBS. The cells were incubated with rabbit anti-PECAM (H-300; 1:50 dilution; Santa Cruz Biotechnology) followed by treatment with Alexa Fluor 488-conjugated anti-rabbit IgG (1:100 dilution; Invitrogen). DAPI was used as a counterstain for nuclei. The samples were mounted in CC/Mount and visualized under the FV1000-D laser scanning confocal microscope (Olympus).

For Western blot analysis, HUVECs were seeded in culture dishes (10 cm). On reaching 30-50% confluence, the cells were transfected in 9 mL of medium containing Lipofectamine RNAiMAX (12 μL, Invitrogen) and PECAM-1 siRNA (72 pmol; Qiagen) and non-targeted siRNA, siControl, for 24 h. After transfection, whole cell proteins were obtained using RIPA buffer containing a protease inhibitor mixture (Roche Applied Science). The lysates (15 μg of proteins) were separated by SDS-PAGE using 4-20% gradient gels and transferred to a nitrocellulose membrane. The membrane was incubated with an anti-PECAM antibody (1:1000 dilution; Santa Cruz Biotechnology) and an anti-GAPDH antibody (1:1000 dilution; Millipore) as a primary antibody at 4 °C overnight. GAPDH was an internal reference. The primary antibodies were detected with horseradish peroxidase-conjugated donkey anti-goat and anti-mouse IgG (1:3000 and 1:1000 dilution, respectively; Jackson ImmunoResearch). The antibody-protein complexes were visualized by a Western Lightning ECL Pro (Perkin Elmer). The protein bands were analyzed using a Luminoimage Analyzer LAS-1000 PLUS (Fuji Film).

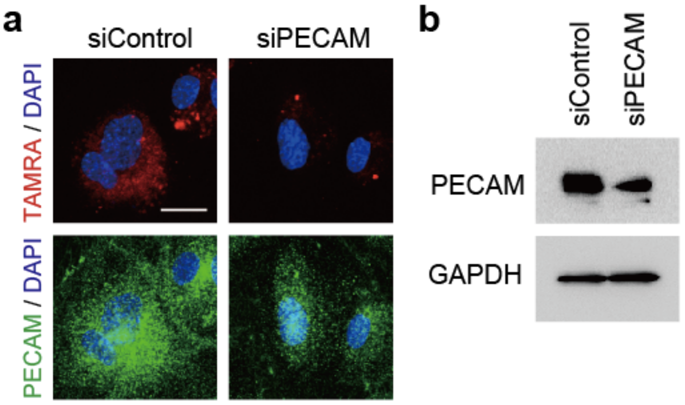

Figure S26. Cell imaging and Western blot after siRNA transfection. a, the pre-targeting imaging was performed using **1a** and **2a** (*red*) after transfection with siRNA against PECAM (siPECAM) or non-targeted siRNA (siControl). The cells were fixed, stained with anti-PECAM (*green*), and DAPI (*blue*). The scale bar indicates 20 μm. **b**, The PECAM knockdown by siRNA transfection was evaluated by Western blot analysis. GAPDH was an internal control.

References

1. Ning, X., Guo, J., Wolfert, M. A., Boons, G. J. Visualizing metabolically labeled glycoconjugates of living cells by copper-free and fast Huisgen cycloadditions. *Angew. Chem. Int. Ed.* **47**, 2253-2255 (2008).
2. Tanaka, K., Siwu, E. R. O., Minami, K., Hasegawa, K., Nozaki, S., Kanayama, Y., Koyama, K., Chen, W. C., Paulson, J. C., Watanabe, Y., Fukase, K. Noninvasive imaging of dendrimer-type N-glycan clusters: in vivo dynamics dependence on oligosaccharide structure. *Angew. Chem. Int. Ed.* **49**. 8195-8200 (2010).

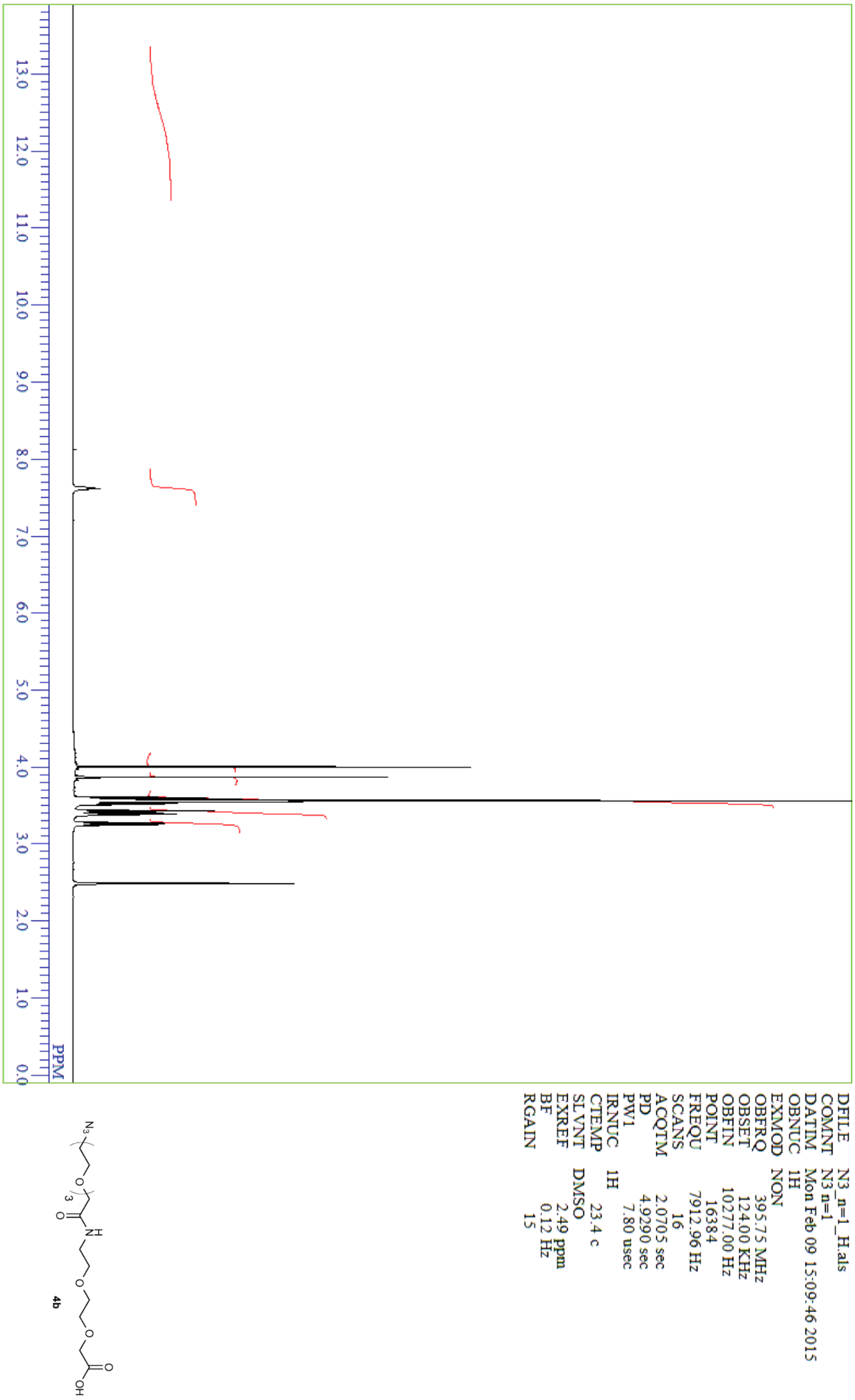
PPM
13.0
12.0
11.0
10.0
9.0
8.0
7.0
6.0
5.0
4.0
3.0
2.0
1.0
0.0
DFILE N3_n=1_H.als
COMNT N3 n=1
DATIM Mon Feb 09 15:09:46 2015
OBNUC 1H
EXMOD NON
OBFRQ 395.75 MHz
OBSET 124.00 KHz
OBFIN 10277.00 Hz
POINT 16384
FREQU 7912.96 Hz
SCANS 16
ACQTM 2.0705 sec
PD 4.9290 sec
PW1 7.80 usec
IRNUC 1H
CTEMP 23.4 c
SLVNT DMSO
EXREF 2.49 ppm
BF 0.12 Hz
RGAIN 15
4b

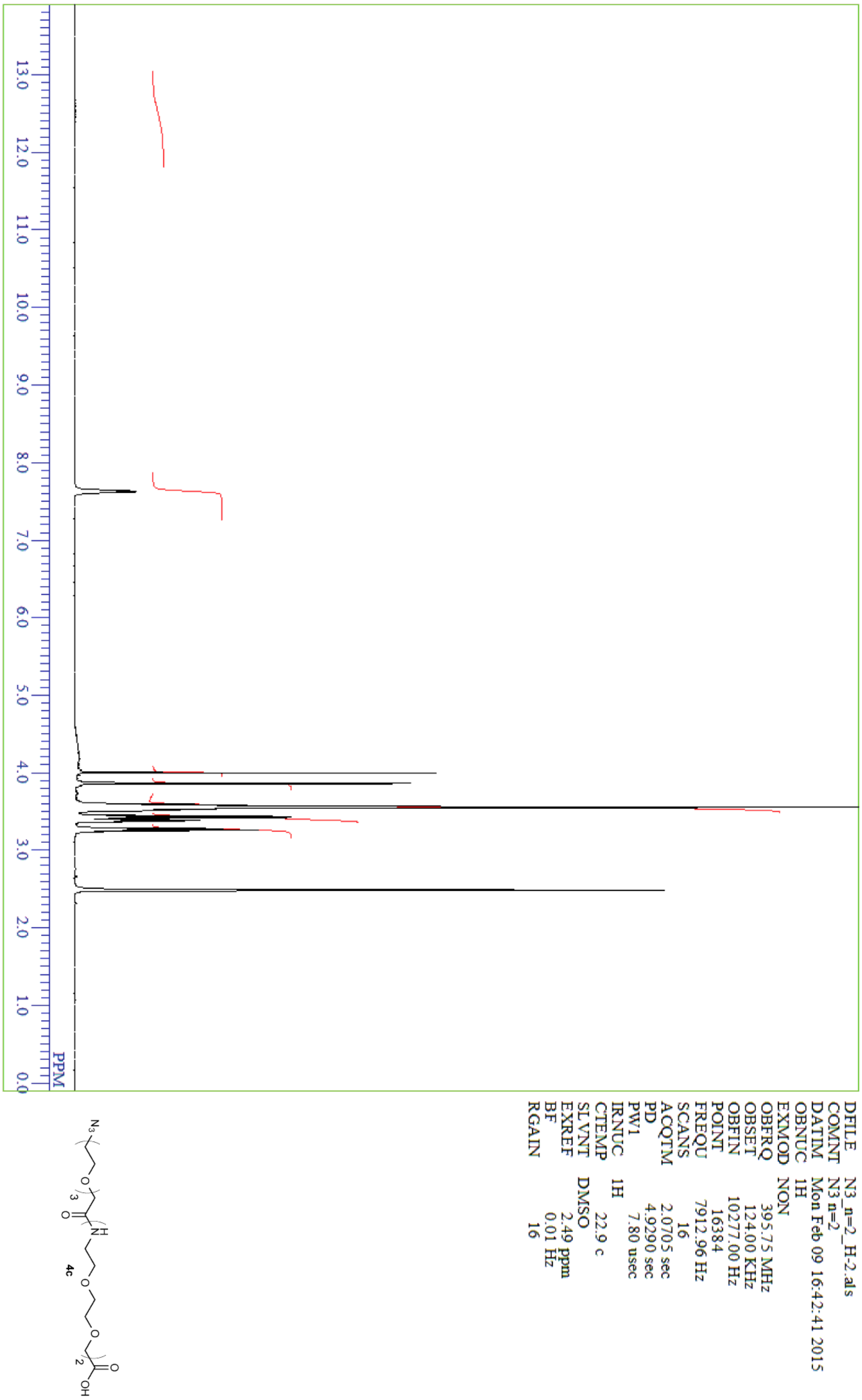
PPM
13.0
12.0
11.0
10.0
9.0
8.0
7.0
6.0
5.0
4.0
3.0
2.0
1.0
0.0
DFILE N3_n=2_H-2.als
COMNT N3 n=2
DATIM Mon Feb 09 16:42:41 2015
OBNUC 1H
EXMOD NON
OBFRQ 395.75 MHz
OBSET 124.00 KHz
OBFIN 10277.00 Hz
POINT 16384
FREQU 7912.96 Hz
SCANS 16
ACQTM 2.0705 sec
PD 4.9290 sec
PW1 7.80 usec
IRNUC 1H
CTEMP 22.9 c
SLVNT DMSO
EXREF 2.49 ppm
BF 0.01 Hz
RGAIN 16
4c

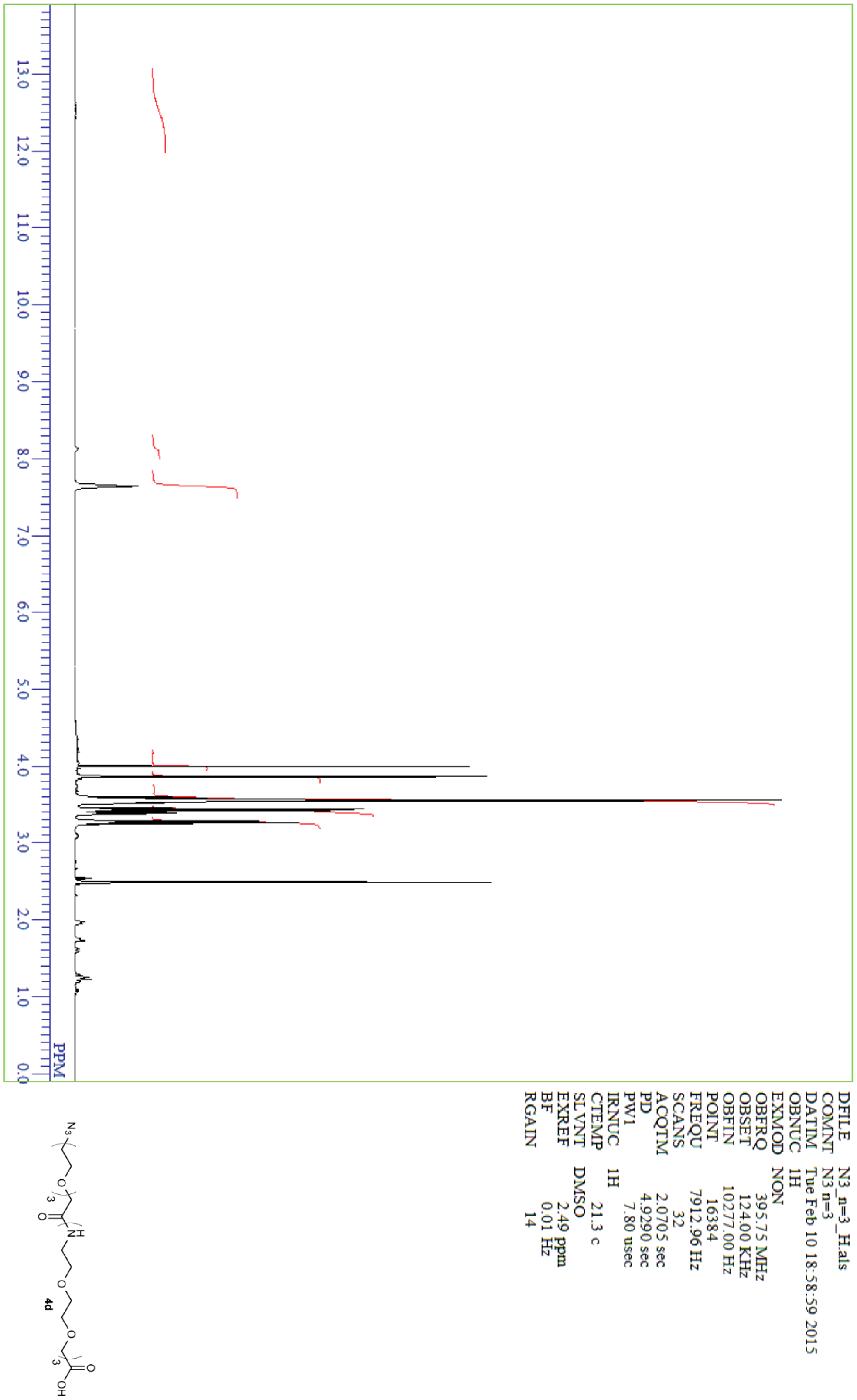
13.0
12.0
11.0
10.0
9.0
8.0
7.0
6.0
5.0
4.0
3.0
2.0
1.0
0.0
PPM
DFILE N3_n=3_H.als
COMNT N3 n=3
DATIM Tue Feb 10 18:58:59 2015
OBNUC 1H
EXMOD NON
OBFRQ 395.75 MHz
OBSET 124.00 KHz
OBFIN 10277.00 Hz
POINT 16384
FREQU 7912.96 Hz
SCANS 32
ACQTM 2.0705 sec
PD 4.9290 sec
PW1 7.80 usec
IRNUC 1H
CTEMP 21.3 c
SLVNT DMSO
EXREF 2.49 ppm
BF 0.01 Hz
RGAIN 14
4d

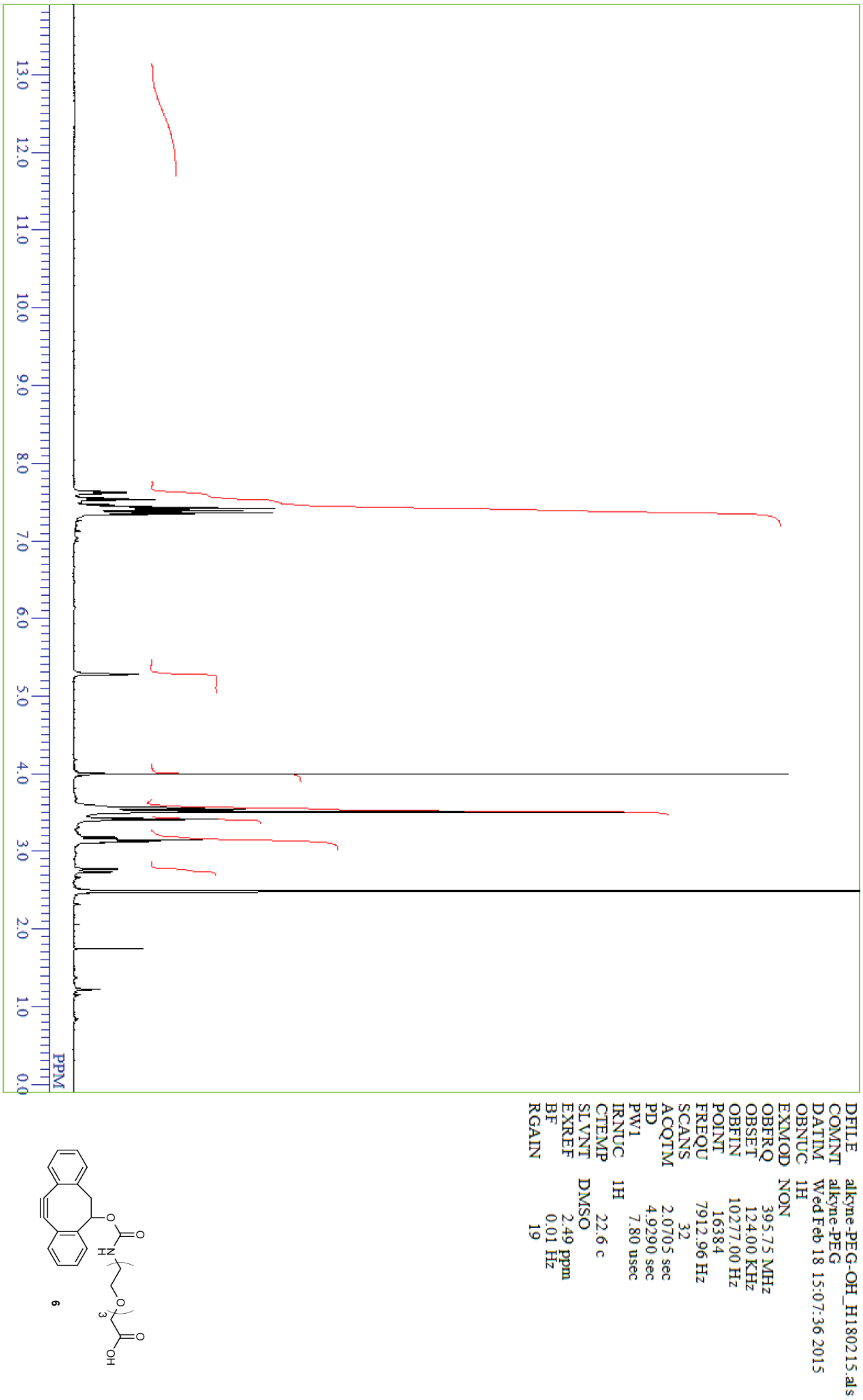

13.0
12.0
11.0
10.0
9.0
8.0
7.0
6.0
5.0
4.0
3.0
2.0
1.0
0.0
PPM
DFILE alkyne-PEG-OH_H180215.als
COMNT alkyne-PEG
DATIM Wed Feb 18 15:07:36 2015
OBNUC 1H
EXMOD NON
OBFRQ 395.75 MHz
OBSET 124.00 KHz
OBFIN 10277.00 Hz
POINT 16384
FREQU 7912.96 Hz
SCANS 32
ACQTM 2.0705 sec
PD 4.9290 sec
PW1 7.80 usec
IRNUC 1H
CTEMP 22.6 c
SLVNT DMSO
EXREF 2.49 ppm
BF 0.01 Hz
RGAIN 19
O
O
N
H
O
3
O
OH

6

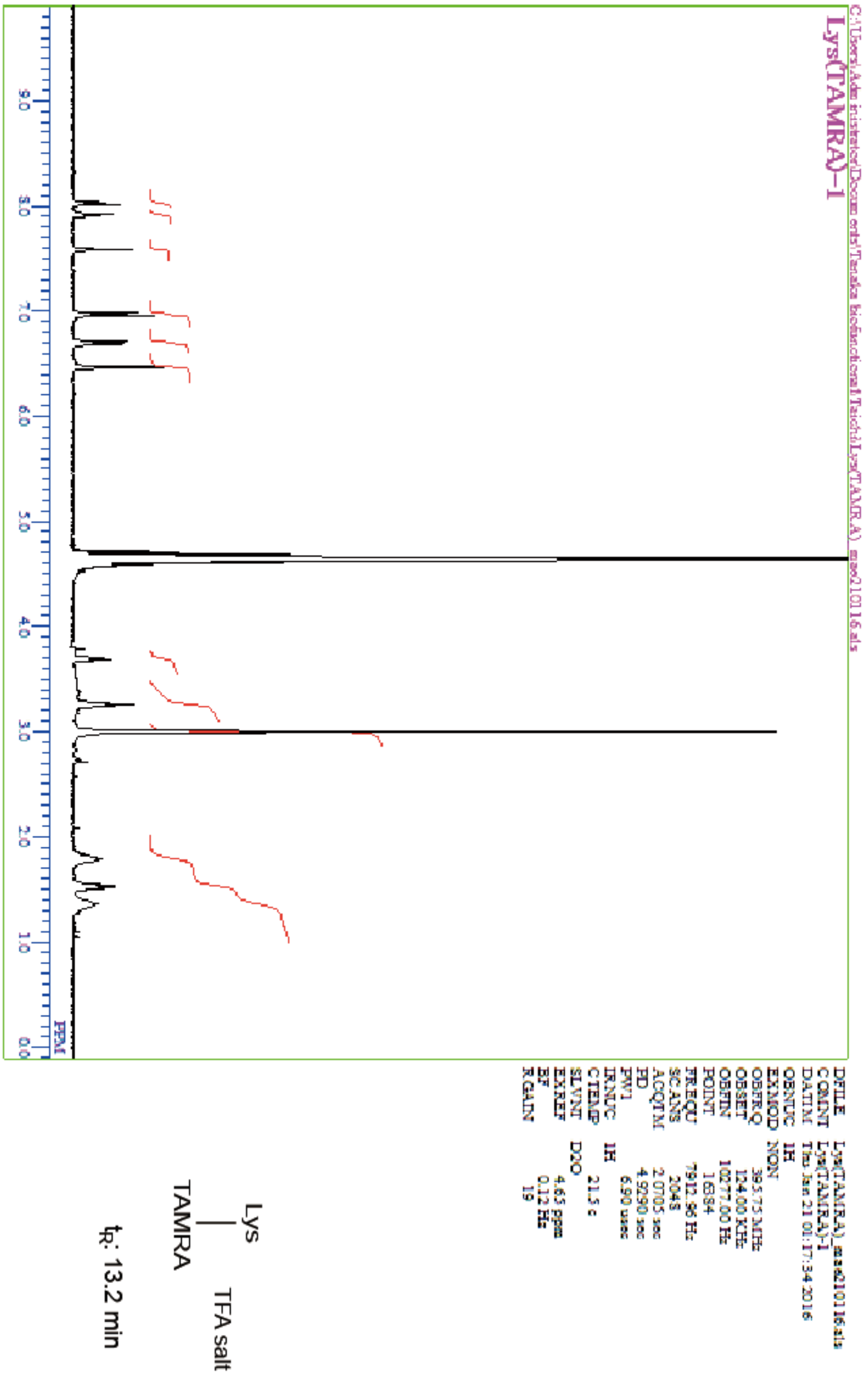
Lys(TAMRA)-1
Lys
TAMRA
TFA salt
tR: 13.2 min

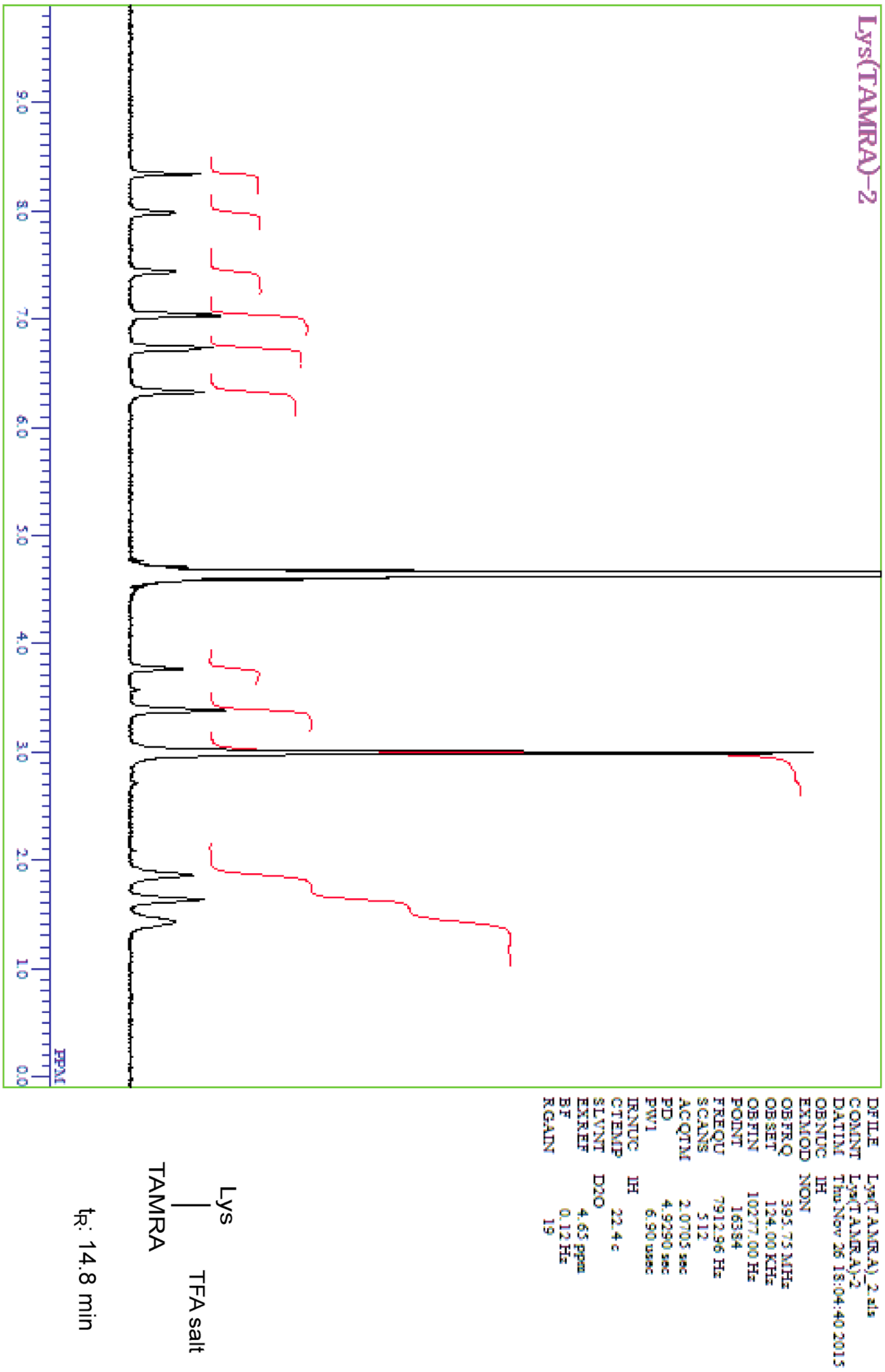
Lys(TAMRA)-2
DFILE Lys(TAMRA)_2.als
COMNT Lys(TAMRA)-2
DATIM Thu Nov 26 18:04:40 2015
OBNUC 1H
EXMOD NON
OBFRQ 395.75 MHz
OBSET 124.00 KHz
OBFIN 10277.00 Hz
POINT 16384
FREQU 7912.96 Hz
SCANS 512
ACQTM 2.0705 sec
PD 4.9290 sec
PW1 6.90 usec
IRNUC 1H
CTEMP 22.4 c
SLVNT D2O
EXREF 4.65 ppm
BF 0.12 Hz
RGAIN 19
PPM
9.0
8.0
7.0
6.0
5.0
4.0
3.0
2.0
1.0
0.0
Lys
TAMRA
TFA salt
tR: 14.8 min

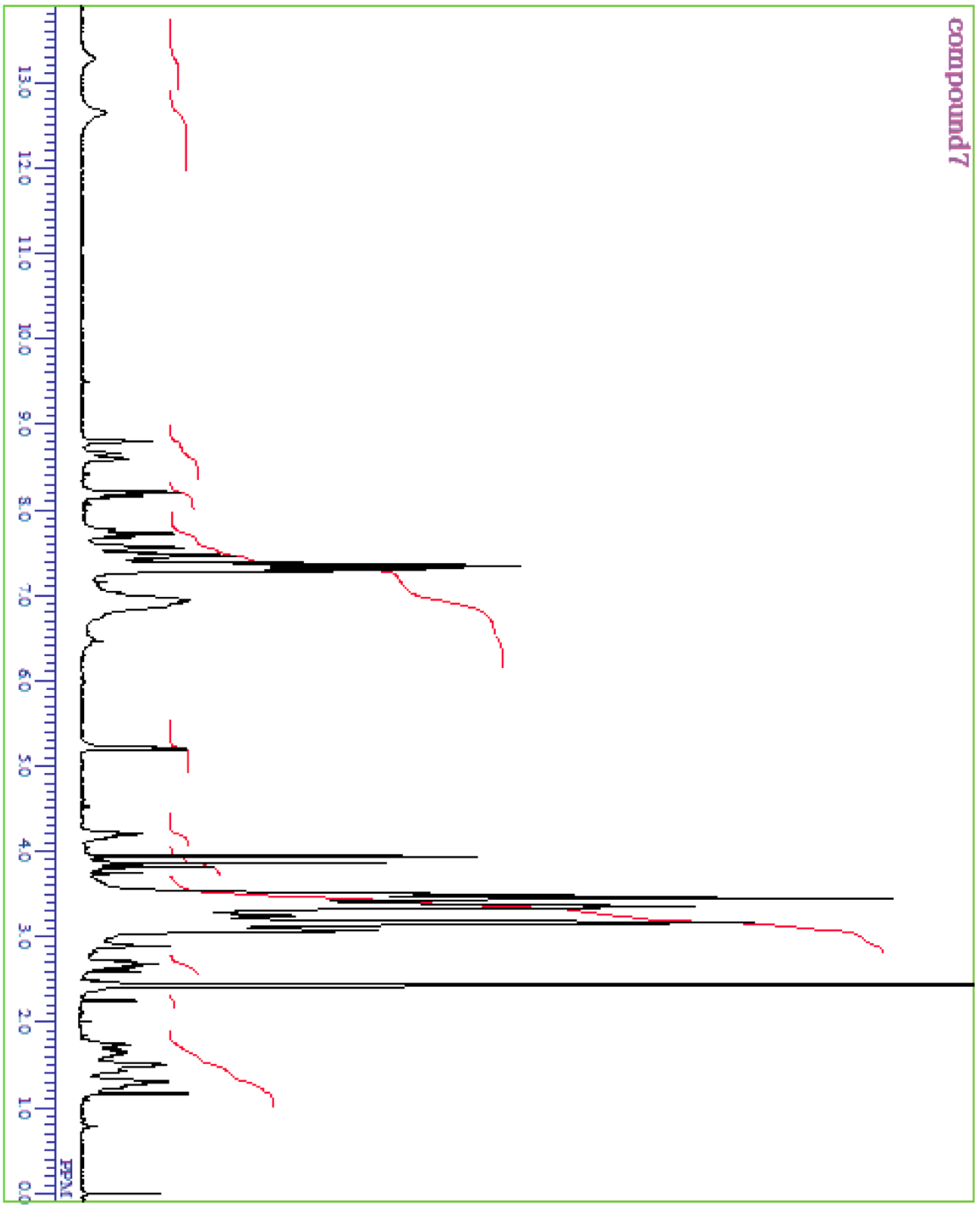
compound7
PPM
13.0
12.0
11.0
10.0
9.0
8.0
7.0
6.0
5.0
4.0
3.0
2.0
1.0
0.0
DFILE acetylene-Lys(TAMRA)-OH_H05111
COMNT compound7
DATIM Thu Nov 05 17:38:24 2015
OBNUC 1H
EXMOD NON
OBFRQ 395.75 MHz
OBSET 124.00 KHz
OBFIN 10277.00 Hz
POINT 16384
FREQU 7912.96 Hz
SCANS 512
ACQTM 2.0705 sec
PD 4.9290 sec
PW1 6.90 usec
IRNUC 1H
CTEMP 21.9 c
SLVNT DMSO
EXREF 0.00 ppm
BF 0.12 Hz
RGAIN 20

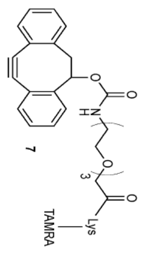
Lys
TAMRA

7

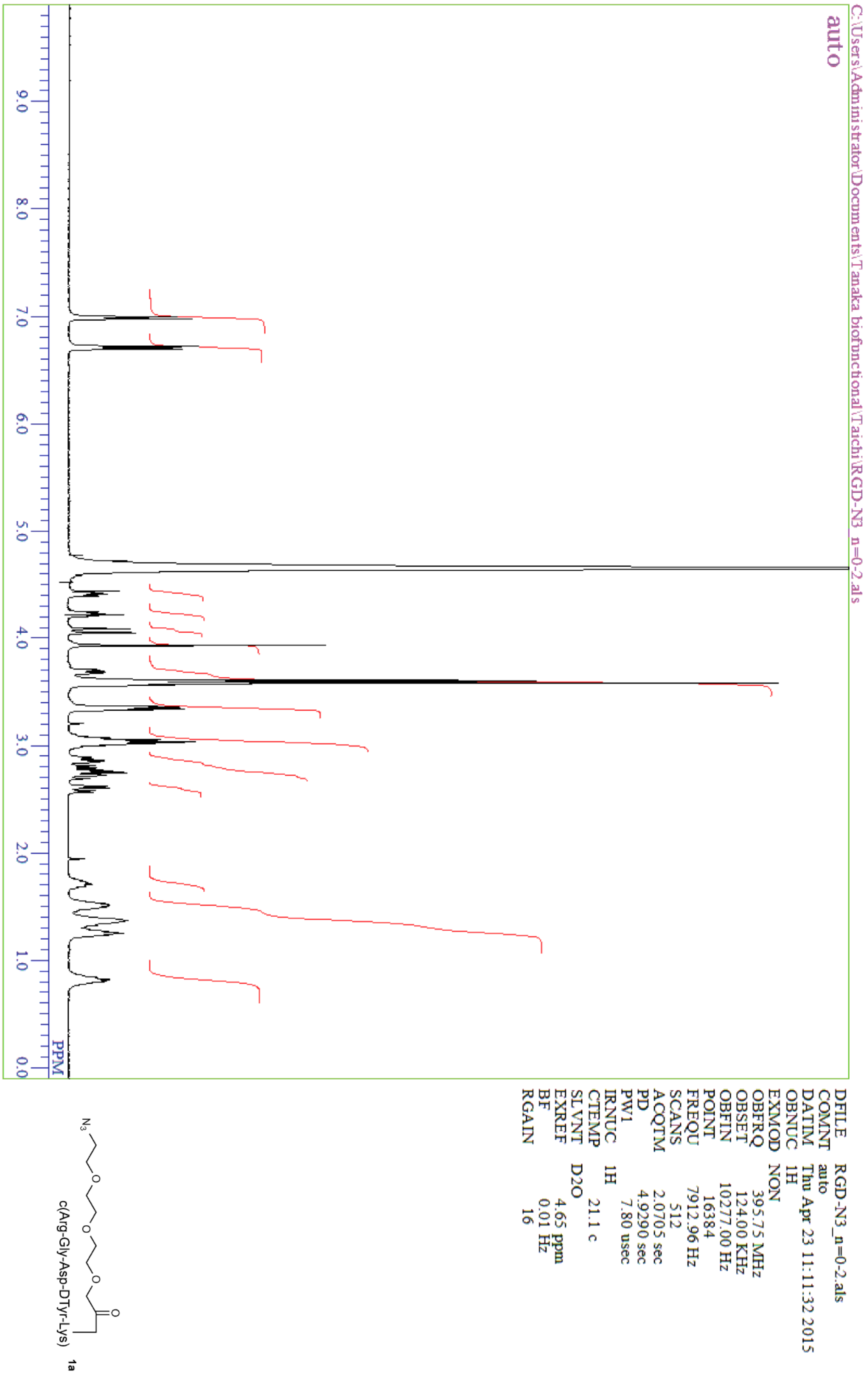
C:\Users\Administrator\Documents\Tanaka biofunctional\Taichi\RGD-N3_n=0-2.als
auto
9.0
8.0
7.0
6.0
5.0
4.0
3.0
2.0
1.0
0.0
PPM
DFILE RGD-N3_n=0-2.als
COMNT auto
DATIM Thu Apr 23 11:11:32 2015
OBNUC 1H
EXMOD NON
OBFRQ 395.75 MHz
OBSET 124.00 KHz
OBFIN 10277.00 Hz
POINT 16384
FREQU 7912.96 Hz
SCANS 512
ACQTM 2.0705 sec
PD 4.9290 sec
PW1 7.80 usec
IRNUC 1H
CTEMP 21.1 c
SLVNT D2O
EXREF 4.65 ppm
BF 0.01 Hz
RGAIN 16
N3
O
O
O
O
c(Arg-Gly-Asp-DTyr-Lys)
1a

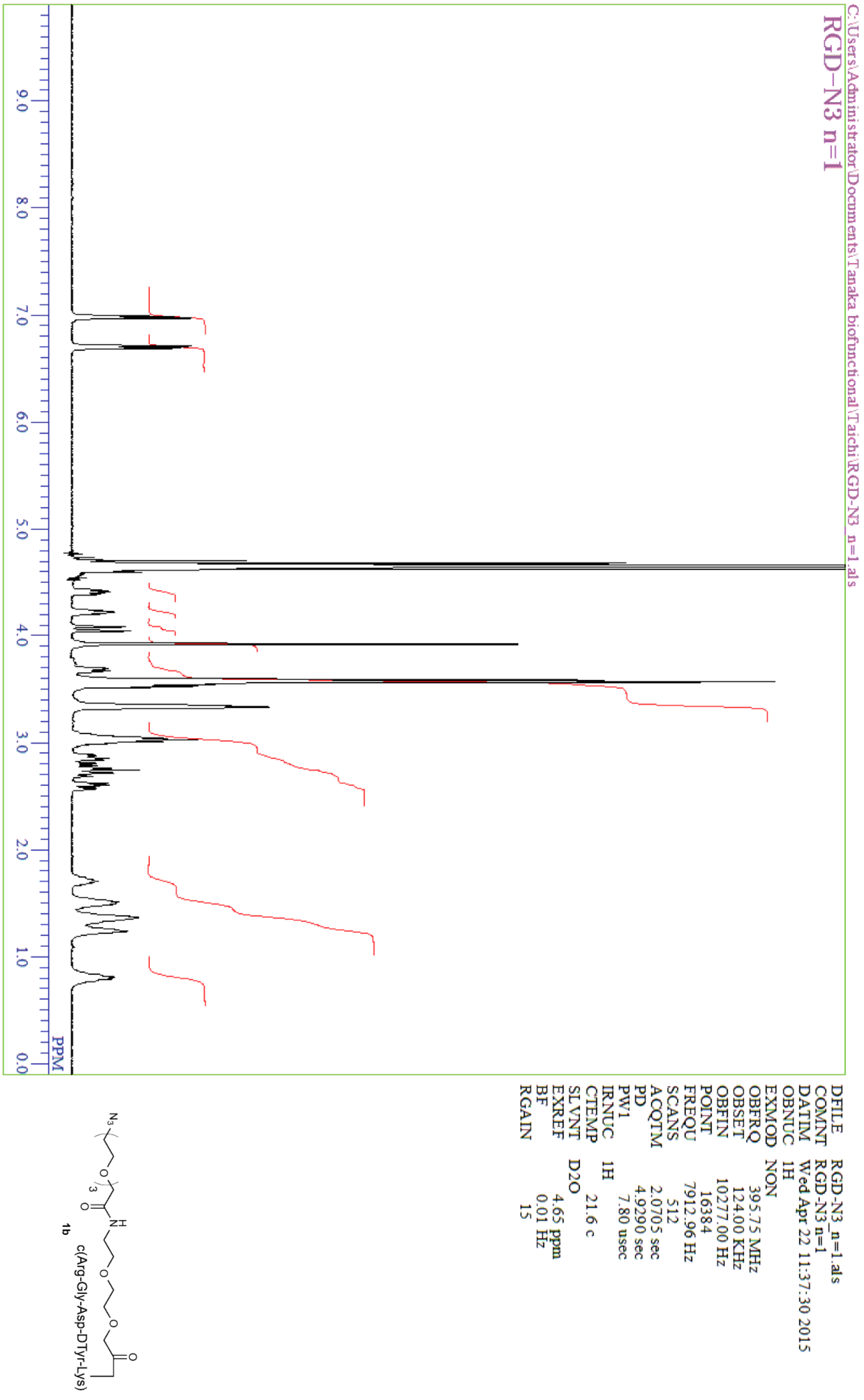
C:\Users\Administrator\Documents\Tanaka biofunctional\Taichi\RGD-N3_n=1.als
RGD−N3 n=1
PPM
9.0 8.0 7.0 6.0 5.0 4.0 3.0 2.0 1.0 0.0
DFILE RGD-N3_n=1.als
COMNT RGD-N3 n=1
DATIM Wed Apr 22 11:37:30 2015
OBNUC 1H
EXMOD NON
OBFRQ 395.75 MHz
OBSET 124.00 KHz
OBFIN 10277.00 Hz
POINT 16384
FREQU 7912.96 Hz
SCANS 512
ACQTM 2.0705 sec
PD 4.9290 sec
PW1 7.80 usec
IRNUC 1H
CTEMP 21.6 c
SLVNT D2O
EXREF 4.65 ppm
BF 0.01 Hz
RGAIN 15
c(Arg-Gly-Asp-DTyr-Lys)
1b

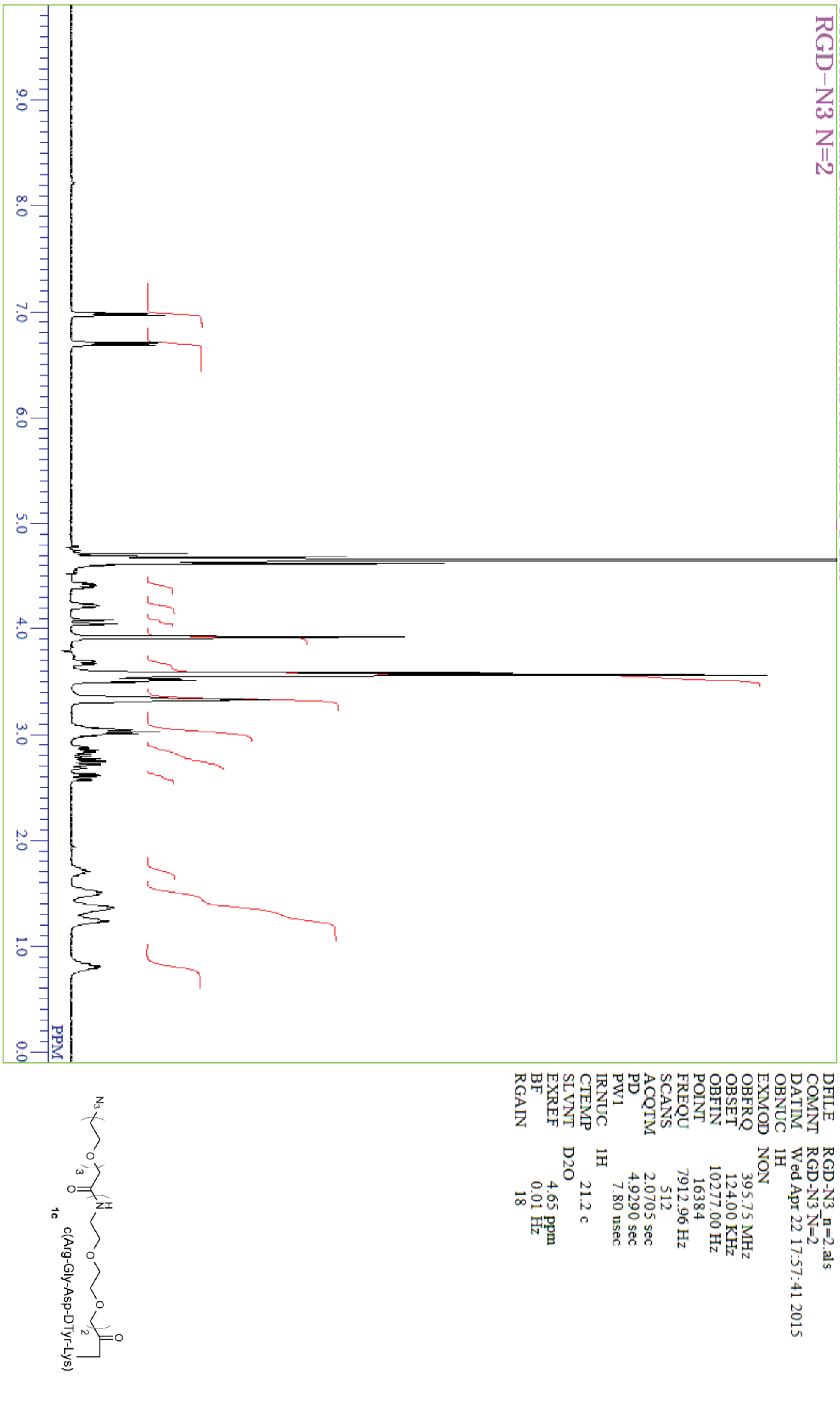
C:\Users\Administrator\Documents\Tanaka biofunctional\Taichi\RGD-N3_n=2.als
RGD−N3 N=2
PPM
9.0
8.0
7.0
6.0
5.0
4.0
3.0
2.0
1.0
0.0
DFILE RGD-N3_n=2.als
COMNT RGD-N3 N=2
DATIM Wed Apr 22 17:57:41 2015
OBNUC 1H
EXMOD NON
OBFRQ 395.75 MHz
OBSET 124.00 KHz
OBFIN 10277.00 Hz
POINT 16384
FREQU 7912.96 Hz
SCANS 512
ACQTM 2.0705 sec
PD 4.9290 sec
PW1 7.80 usec
IRNUC 1H
CTEMP 21.2 c
SLVNT D2O
EXREF 4.65 ppm
BF 0.01 Hz
RGAIN 18
c(Arg-Gly-Asp-DTyr-Lys)
1c

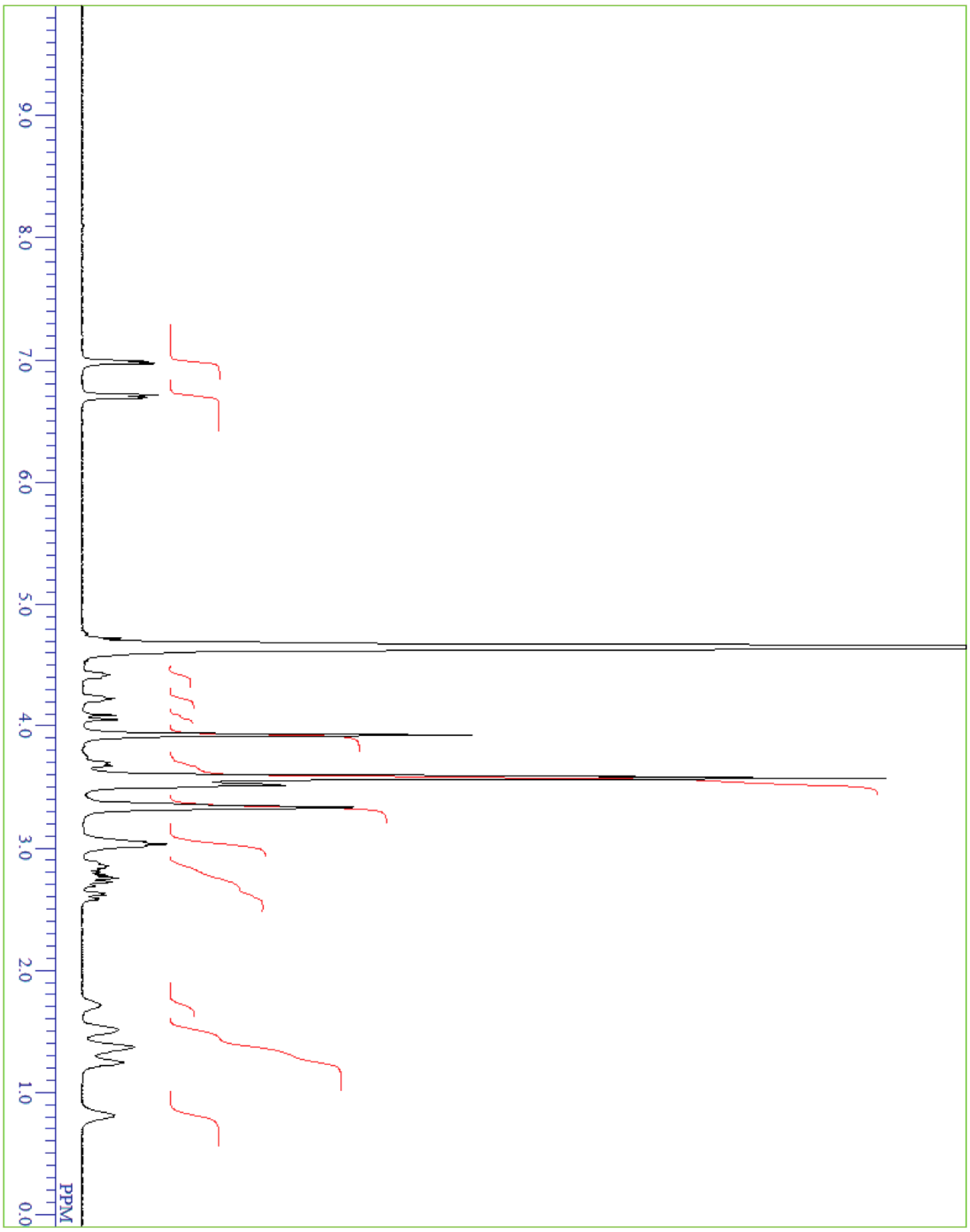

9.0
8.0
7.0
6.0
5.0
4.0
3.0
2.0
1.0
0.0
PPM
DFILE RGD-N3_n=3.als
COMNT RGD-N3 n=3
DATIM Mon May 04 17:29:50 2015
OBNUC 1H
EXMOD NON
OBFRQ 395.75 MHz
OBSET 124.00 KHz
OBFIN 10277.00 Hz
POINT 16384
FREQU 7912.96 Hz
SCANS 256
ACQTM 2.0705 sec
PD 4.9290 sec
PW1 7.80 usec
IRNUC 1H
CTEMP 21.5 c
SLVNT D2O
EXREF 4.65 ppm
BF 0.01 Hz
RGAIN 14

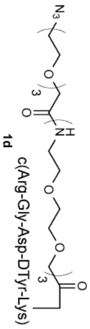

c(Arg-Gly-Asp-DTyr-Lys)

1d

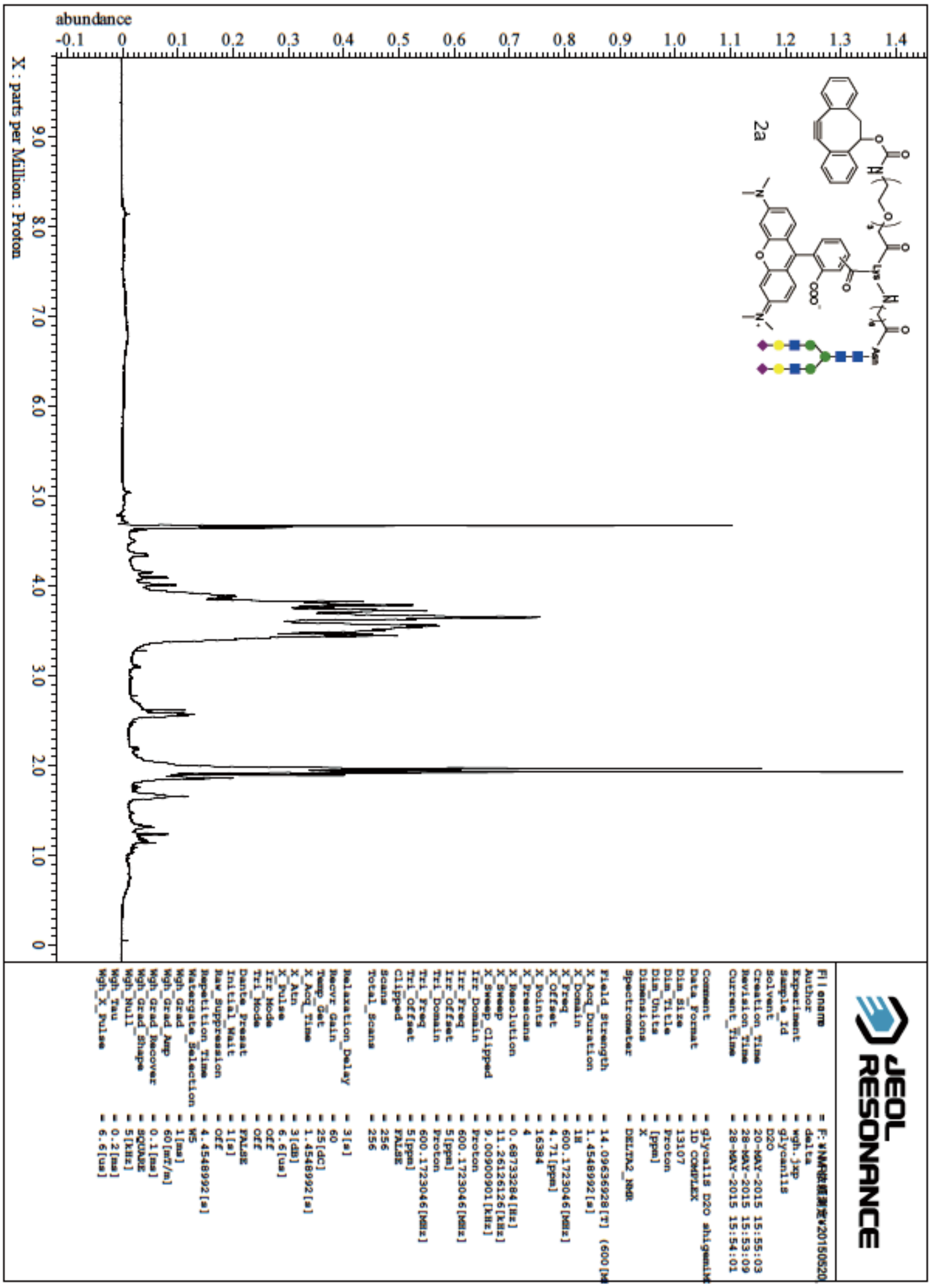
abundance
X : parts per Million : Proton
2a
JEOL RESONANCE

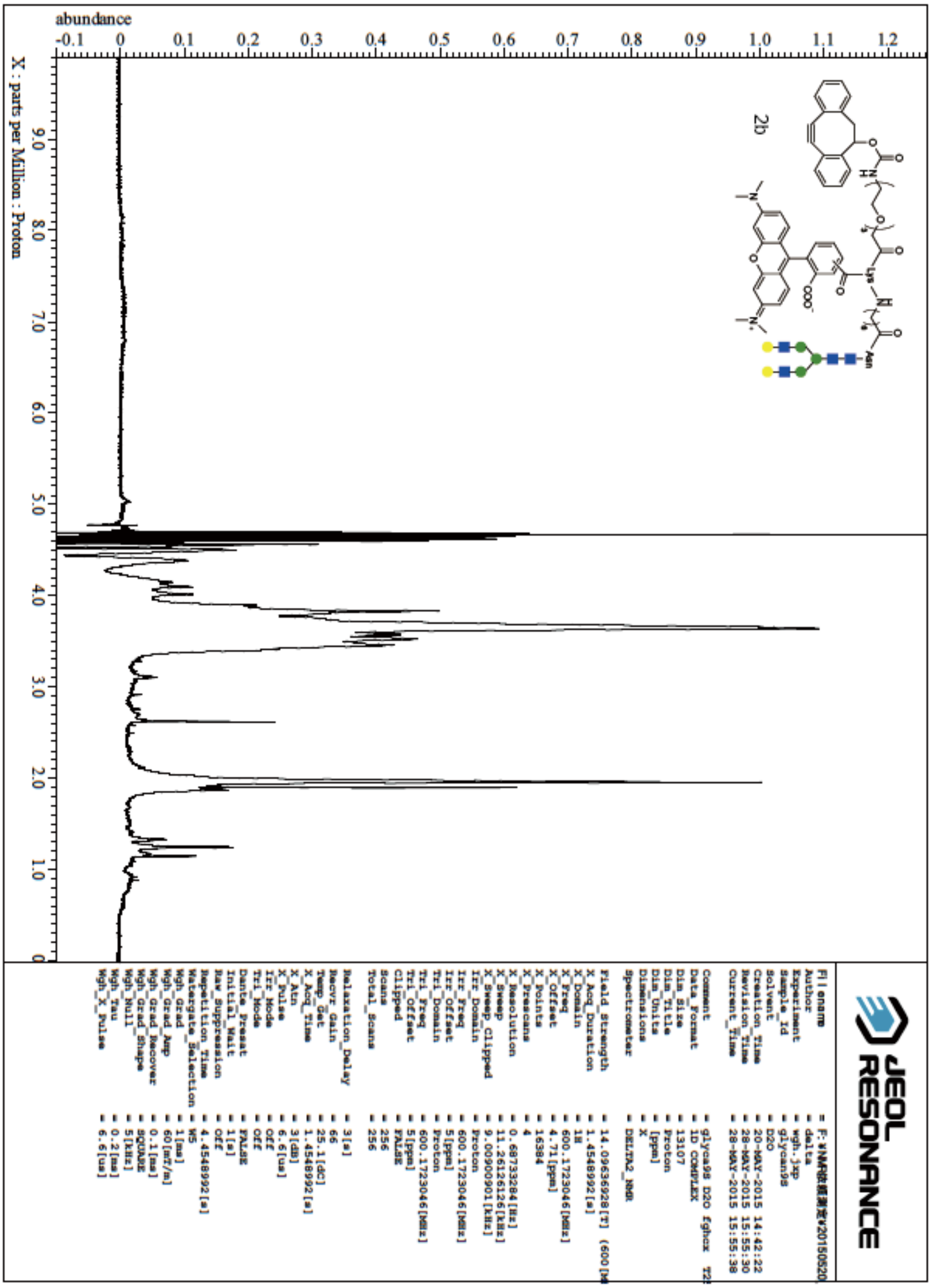
abundance
X : parts per Million : Proton
2b
JEOL RESONANCE
